# The impact of chromosomal fusions on 3D genome folding and recombination in the germ line

**DOI:** 10.1038/s41467-021-23270-1

**Published:** 2021-05-20

**Authors:** Covadonga Vara, Andreu Paytuví-Gallart, Yasmina Cuartero, Lucía Álvarez-González, Laia Marín-Gual, Francisca Garcia, Beatriu Florit-Sabater, Laia Capilla, Rosa Ana Sanchéz-Guillén, Zaida Sarrate, Riccardo Aiese Cigliano, Walter Sanseverino, Jeremy B. Searle, Jacint Ventura, Marc A. Marti-Renom, François Le Dily, Aurora Ruiz-Herrera

**Affiliations:** 1 Departament de Biologia Cellular, Fisiologia i Immunologia, Universitat Autònoma de Barcelona, Cerdanyola del Vallès, Spain; 2 Genome Integrity and Instability Group, Institut de Biotecnologia i Biomedicina, Universitat Autònoma de Barcelona, Cerdanyola del Vallès, Spain; 3 Sequentia Biotech, Barcelona, Spain; 4 Centre for Genomic Regulation, The Barcelona Institute for Science and Technology, Barcelona, Spain; 5 CNAG-CRG, Centre for Genomic Regulation, The Barcelona Institute of Science and Technology, Barcelona, Spain; 6 Servei de Cultius Cel.lulars, Universitat Autònoma de Barcelona, Cerdanyola del Vallès, Spain; 7 Departament de Biologia Animal, Biologia Vegetal i Ecologia, Universitat Autònoma de Barcelona, Cerdanyola del Vallès, Spain; 8 Department of Ecology and Evolutionary Biology, Corson Hall, Cornell University, Ithaca, NY USA; 9 Pompeu Fabra University, Barcelona, Spain; 10 ICREA, Pg. Lluís Companys 23, Barcelona, Spain; 11 Present Address: Instituto de Ecología AC (INECOL), Red de Biología Evolutiva, Xalapa, Veracruz, Mexico

**Keywords:** Chromosomes, Genome

## Abstract

The spatial folding of chromosomes inside the nucleus has regulatory effects on gene expression, yet the impact of genome reshuffling on this organization remains unclear. Here, we take advantage of chromosome conformation capture in combination with single-nucleotide polymorphism (SNP) genotyping and analysis of crossover events to study how the higher-order chromatin organization and recombination landscapes are affected by chromosomal fusions in the mammalian germ line. We demonstrate that chromosomal fusions alter the nuclear architecture during meiosis, including an increased rate of heterologous interactions in primary spermatocytes, and alterations in both chromosome synapsis and axis length. These disturbances in topology were associated with changes in genomic landscapes of recombination, resulting in detectable genomic footprints. Overall, we show that chromosomal fusions impact the dynamic genome topology of germ cells in two ways: (i) altering chromosomal nuclear occupancy and synapsis, and (ii) reshaping landscapes of recombination.

## Abstract (web summary)

How mammalian genomes are packaged and the heritability of structural variations in genome folding is incomplete. Here, the authors investigate the impact of chromosomal fusions on three-dimensional genome topology and meiotic recombination, highlighting the implications of large-scale genome reorganizations on genome function, evolution, and fertility.

## Introduction

Higher-order chromatin structure demarcates the limits of gene-regulatory domains^1–3^. Thus, disturbances of domain architecture due to genome reshuffling (i.e., inversions, fusions, or indels) represent a nongradual change in gene regulation because shifting of domain boundaries exposes genes to novel regulatory environments^4^. Models^5,6^ and growing experimental evidence^7,8^ suggest that indels and inversions can alter interactions between contiguous topological associated domains (TADs), which can lead to oncogene activation, morphological alterations, and novel gene functions. However, the impact of balanced chromosomal rearrangements, such as Robertsonian (Rb) fusions^9^, on genome architecture and its heritability are less explored. This is of particular relevance since Rb fusions represent the most common chromosomal rearrangement in nature (from plants to mammals)^10^, and are linked to recurrent miscarriages, infertility, and aneuploid offspring in humans^11^. In fact, it has long been suggested, although not yet empirically demonstrated at the genome level, that the presence of chromosomal fusions in the germ line can alter segregation patterns (the so-called interchromosomal effect^12^).

Germ cells are a unique cell model to test the genome-wide impact of chromosomal fusions—they have sequential developmental stages that involve dramatic and tightly regulated chromosomal movements and chromatin remodeling. These include changes in intra-/interchromosomal interaction ratios, distance-dependent interaction frequencies, genomic compartments, TADs, occupancy of insulator proteins (CTCF and cohesins), and gene expression^13–16^. The delicate fine-tuning between chromatin remodeling, architectural proteins, and cell-specific gene expression is crucial during the first meiotic prophase (prophase I) when homologous chromosomes align, pair, synapse, and recombine^16^.

Recombination has a dual role in sexual reproduction: (i) it assembles new combinations of allelic variants, contributing to the maintenance of genetic diversity, and (ii) establishes physical associations between homologous chromosomes that enable faithful chromosomal segregation during meiosis. Importantly, recombination can be modulated not only by factors that control the formation of meiotic crossovers (COs) during early meiosis (e.g., chromosome axis length is determined by chromatin loop length^17–19^) but also by large-scale structural reorganizations that can dramatically alter the genomic landscape^20–22^. Yet, the impact of large-scale genome reshuffling (e.g., chromosomal fusions) on the three-dimensional genome topology in germ cells and its implications for recombination remain unknown.

Here, we take advantage of chromosome conformation capture followed by deep sequencing (Hi-C) in combination with cytological analysis of CO events and SNP genotyping to study how genome folding and recombination landscapes are affected by chromosomal fusions in the mammalian germ line. We analyzed wild Western European house mice (*Mus musculus domesticus*) from the northeast of the Iberian Peninsula, belonging to the so-called Barcelona Rb system (BRbS) characterized by a recent evolutionary origin of chromosomal fusions^21,23,24^. The standard karyotype of *M. m. domesticus* consists of 40 acrocentric chromosomes, in contrast to BRbS mice, which have a variety of diploid numbers (2*n*) ranging from 2*n* = 27 to 2*n* = 40^23^. This system is characterized by the presence of different Rb fusions distributed in nongeographically coincident clines, leading to a progressive reduction in diploid numbers toward the center of the range^23^. This natural model permits interrogation of the impact that Rb fusions have on chromatin remodeling and fine-scale recombination in the germ line. In particular, we studied how chromosomal fusions alter the nuclear architecture at different hierarchical levels in meiotic (i.e., primary spermatocytes) and postmeiotic cells (i.e., round spermatids), and discuss the implications for evolution and fertility.

## Results

### Variation in recombination rates in natural populations of house mice

**Fig. 1:**
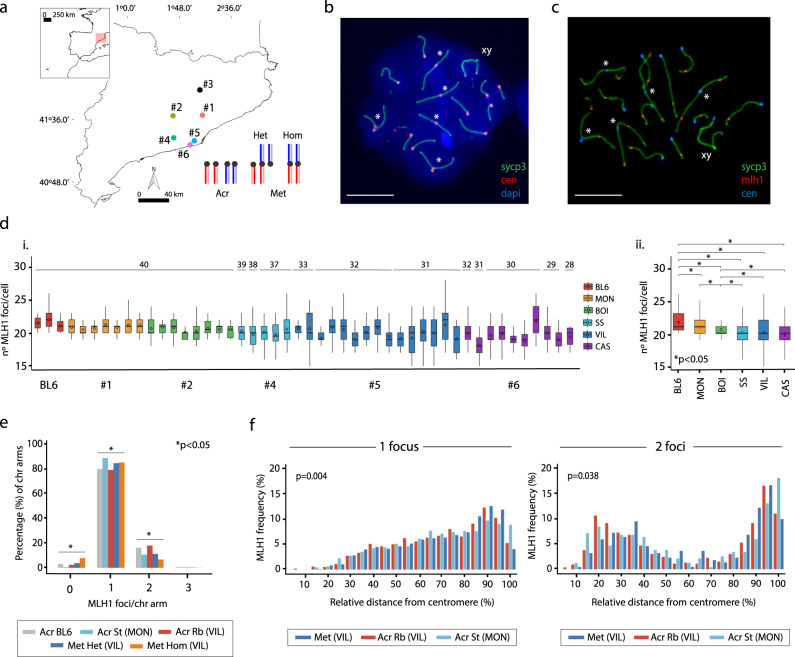
Genomic landscapes of recombination. **a** BRbS populations sampled. See Supplementary Table 1 for the population’s number assignment. The diagram represents different types of chromosomal fusions. Chromosome type legend: Acr, all-acrocentric chromosomes of standard mice; Met Het, Rb chromosomes in heterozygous state of Rb mice; Met Hom, Rb chromosomes in homozygous state of Rb mice. **b** Immunofluorescence of a spermatocyte at pachytene stage from a Rb mouse (2*n* = 32): SYCP3 (green), centromeres (red), and DNA (DAPI, blue). Asterisks indicate Rb chromosomal fusions. Sex chromosomes (XY) are indicated. Scale bar = 10 µm. Immunofluorescence replicates, *n* = 3. **c** Immunofluorescence of a spermatocyte at pachytene stage: SYCP3 (green), MLH1 (red), and centromeres (blue). Asterisks indicate Rb chromosomal fusions. Sex chromosomes (XY) are indicated. Scale bar = 10 µm. Immunofluorescence replicates, *n* = 3. **d** Boxplots depicting the number of MLH1 foci/cell per specimen represented (i) individually (colors correspond to panel **a**) and (ii) per population. Boxplots are presented as mean values ± SD; center line, median; center diamond, mean. (i) Three laboratory mice (BL6) are included for comparison. Boxplots indicating mean values and standard deviations are shown for each individual. BRbS populations: CAS Castelldefels, BOI Castellfollit del Boix, MON Caldes de Montbuí, SS Sant Sadurní d’Anoia, VIL Viladecans. The diploid number of each mouse specimen is indicated on top of each boxplot. Source data are provided as a Source Data file. (ii) Mean numbers of MLH1 foci/cell in laboratory mice (BL6) and wild-caught standard (St) and Rb mice represented per population. *P* values (Wilcoxon’s rank-sum test followed by Dunn’s tests (two-sided) adjusted by Bonferroni, *P < 0.05) represent differences between populations (BL6, laboratory mice (*n* = 55 cells); CAS (*n* = 296 cells), BOI (*n* = 174 cells), MON (*n* = 174 cells), SS (*n* = 206 cells), VIL (*n* = 605 cells)). Source data are provided as a Source Data file. **e** Percentage of chromosomal arms showing the different number (0, 1, 2, or 3) of MLH1 foci per-chromosome (*χ*^2^ test, one-sided, *P < 0.05). The number of chromosome arms analyzed per arm type: *n* = 1046 for Acr BL6; *n* = 1140 for Acr St (MON); *n* = 2233 for Acr Rb (VIL); *n* = 483 for Met Het (VIL); *n* = 1517 for Met Hom. Source data are provided as a Source Data file. **f** Distribution of MLH1 foci along individual chromosomal arms with one (left panel) or two (right panel) MLH1 foci from panel **e**. The *X* axis represents the relative positions on the chromosomal axes from the centromere (0%) to the distal telomere (100%) (*χ*^2^ test, one-sided, *P* < 0.05). The *Y* axis indicates the frequency of MLH1 foci in each interval of chromosome arm length. Chromosome type legend: Acr St, all-acrocentric chromosomes of standard mice from MON population (*n* = 1014 chromosomal arms with 1 CO; *n* = 120 chromosomal arms with 2 COs); Acr Rb, acrocentric chromosomes of Rb mice from VIL population (*n* = 1232 chromosomal arms with 1 CO; *n* = 271 chromosomal arms with 2 COs); Met, arms of metacentric chromosomes of Rb mice from VIL population (*n* = 1272 chromosomal arms with 1 CO; *n* = 112 chromosomal arms with 2 COs). Source data are provided as a Source Data file.

**Fig. 2:**
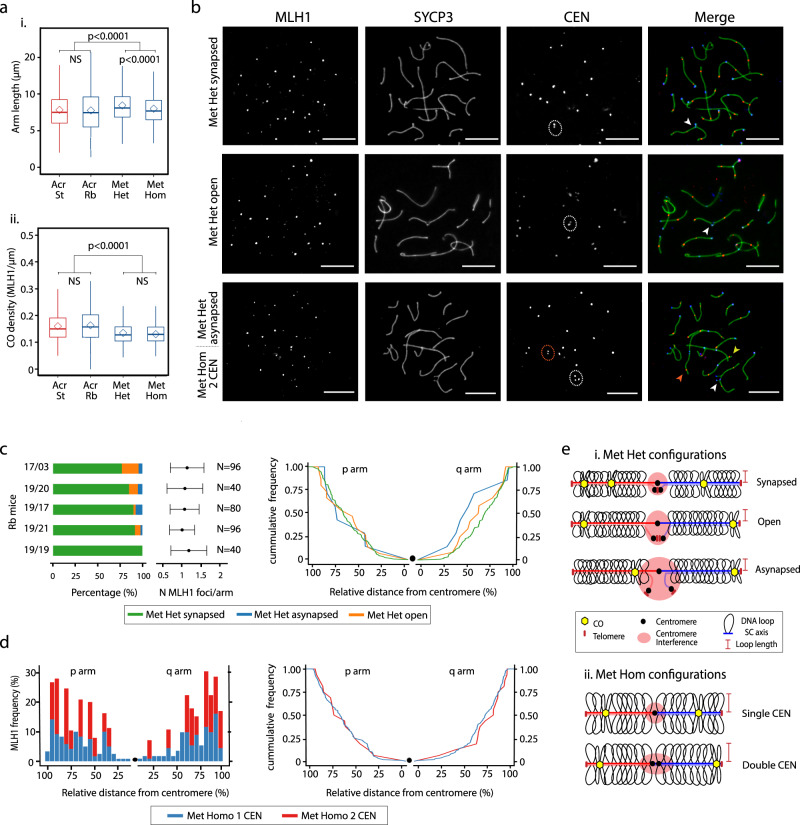
Effect of Rb fusions on recombination and synaptonemal complex length. **a** (i) Axis length (expressed as μm) analysis in standard (St) and Robertsonian (Rb) mice from BRbS (see text and Supplementary Table 1 for further details) according to chromosome types (Dunn’s test, two-sided; *P* < 0.001; NS nonsignificant). Boxplots are presented as mean values ± SD; center line, median; center diamond, mean. Source data are provided as a Source Data file. (ii) Analysis of CO density in the different chromosome types (Mann–Whitney test, two-sided; *P* < 0.0001). Boxplots are presented as mean values ± SD; center line, median; center diamond, mean. Source data are provided as a Source Data file. Chromosome type legend for panels (i) and (ii): Acr, all-acrocentric chromosomes of standard mice (*n* = 1140 chromosomal arms); Acr, Rb all-acrocentric chromosomes of Rb mice (*n* = 1711 chromosomal arms) Met Het, Rb chromosomes in heterozygous state of Rb mice (*n* = 396 chromosomal arms); Met Hom, Rb chromosomes in homozygous state of Rb mice (*n* = 1294 chromosomal arms). **b** Immunofluorescence of primary spermatocytes of Rb mice at the pachynema stage, labeling the synaptonemal complex with SYCP3 (green), the centromeres with CEN (blue), and MLH1 (red). Immunofluorescence replicates, *n* = 3. White dashed circles: centromeric signals in heterozygous fusions. Red dashed: double-centromere signals in homozygous fusions. White arrowheads: Met Het chromosomes. Red arrowhead: Met Hom with double-centromeric signals. Yellow arrowhead: Met Hom with a single centromeric signal. Scale bar = 10 µm. **c** Synapsis and recombination patterns found in mice with Rb fusions a heterozygous state (see Supplementary Table 1 for mouse codes). Left panel: Percentage of heterozygous chromosomes according to synapsis pattern (synapsed, asynapsed, and open) for each of the Rb mice analyzed. Numbers on the left refer to mouse ID (see Supplementary Table 1). Middle panel: Representation of the mean number of MLH1 per arm corresponding to the mice analyzed. *N* = number of cells analyzed per individual. Right panel: Cumulative frequencies of MLH1 distributions in heterozygous metacentrics according to synapsis pattern (synapsed, asynapsed, and open). Source data are provided as a Source Data file. **d** Double-centromeric signals and recombination. Left panel: Distribution of MLH1 foci along individual chromosomal arms in homozygous metacentrics (Met Hom) with a single (1 CEN, blue) or double (2 CEN, red) centromeric signal. The *X* axis represents the positions on the chromosomal axes from the centromeric end (black dot) to the distal telomere. The *Y* axis indicates the frequency of MLH1 foci for each 10% interval of chromosomal length. Right panel: Cumulative frequencies of MLH1 distributions in homozygous metacentrics with a single- or a double-centromeric signal. Source data are provided as a Source Data file. **e** Diagram depicting chromosomal axis (SC) length, DNA loops, and crossovers (COs) observed in fused chromosomes at pachytene in Rb mice. Heterozygous metacentrics (Met Het) chromosomes can occur in three states: synapsed, open, and asynapsed. COs are closer to the centromere in asynapsed chromosomes. Open chromosomes have distal COs, whereas synapsed chromosomes can have more than two COs per arm and in interstitial-to-distal positions. Regardless of the synapsis state, heterozygous metacentrics (Met Het) chromosomes have longer axes than homozygous metacentrics (Met Hom) chromosomes. Conversely, DNA loop lengths in Met Het chromosomes are shorter than in Met Hom chromosomes.

We analyzed the variation in recombination rates in wild-caught BRbS mice and the potential impact of Rb fusions on these patterns. We conducted an integrative approach that combined the cytological mapping of CO events directly in male germ cells (reflecting recombination at the Mbp scale) (Figs. 1, 2 and Supplementary Fig. 1) together with estimates of linkage disequilibrium based on SNP genotyping (recombination at the kbp scale) (Supplementary Fig. 2).

We first experimentally determined the number (frequency) of COs along chromosomal axes in 45 wild mice by the immunodetection of the recombination protein MLH1 (a marker of COs) on pachytene chromosomes (Fig. 1a–c and Supplementary Table 1) (see “Methods”). We also included three laboratory mice (strain C57BL/6J, BL6) for comparison. The CO survey in wild-caught mice included 15 all-acrocentric individuals (two standard populations, 2*n* = 40) and 30 mice with Rb fusions (three populations, 2*n* = 39–28), allowing for microscopic visualization of a total of 1468 spermatocytes (Fig. 1b–d and Supplementary Table 1). Of Rb mice, the population Sant Sadurní d’Anoia (SS) was characterized by a low number (from one to three) of Rb fusions (2*n* = 39–37), whereas the Castelldefels (CAS) and Viladecans (VIL) populations have from four to six Rb fusions (2*n* = 33–28) (Fig. 1d and Supplementary Table 1).

The observed average number of COs ranged from 20.16 (±1.18) MLH1 foci per cell to 21.32 (±1.25) in wild-caught standard mice, and from 18.13 (±1.78) to 21.82 (±2.21) in Rb mice (Fig. 1d). A population-level analysis of COs showed that mice from Rb populations with four or more fusions (CAS and VIL populations) showed greater interindividual variability regarding the number of MLH1 foci numbers per cell (Fig. 1d). In fact, mean numbers of COs per cell were positively correlated with diploid numbers (Spearman, *P* < 0.001), and therefore negatively with the number of Rb fusions (Spearman, *P* < 0.0001) (Supplementary Fig. 1a). Despite fewer CO events, Rb mice showed significantly more interindividual CO covariation than standard mice (Mann–Whitney test, *P* < 0.0001) (Supplementary Fig. 1b). We detected statistical differences in mean numbers of COs per cell among populations, including the wild-caught standard populations MON (Caldes de Montbuí) and BOI (Castellfollit del Boix) and the different Rb populations (Fig. 1d). Overall, mice with Rb fusions (SS, VIL, and CAS populations) showed significantly lower mean numbers of COs per cell (20.09 ± 1.88, 20.12 ± 2.16, and 19.74 ± 1.87) when compared to wild-caught standard (MON and BOI populations) (20.95 ± 1.21 and 20.56 ± 1.37) and BL6 mice (21.67 ± 1.52) (Wilcoxon test, *P* < 0.05) (Fig. 1d), hence confirming previous surveys conducted on different European Rb systems of the house mice^21,25^.

Mirroring descriptions from other mammals^18,19^, both the mean numbers of COs and meiotic double-strand breaks (DSBs) (here exemplified as RAD51 foci) were correlated in both standard (BL6 and wild-caught mice) and Rb mice (Spearman, *P* < 0.05, Supplementary Fig. 1c). The variation in RAD51 foci numbers between laboratory mice and BRbS mice is expected based on CO counts, as these two processes are interrelated^18,19^. As such, differences in DSB numbers between standard and Rb mice, together with the observation of a significantly high CO/DSB ratio in Rb mice (Supplementary Fig. 1d), suggest that a higher proportion of DSBs are resolved as COs when compared to standard mice (Supplementary Fig. 1e).

### Chromosomal fusions reshape genomic landscapes of recombination

Since each pair of homologous chromosomes requires one CO to ensure proper chromosomal segregation (and hence fertility) during the first meiotic division (the so-called obligatory CO^26^), we quantitatively assessed whether the number of COs per chromosomal arm (0, 1, 2, or 3) was altered in Rb mice when compared to wild-caught standard and laboratory mice. To that aim and in order to reduce the intrinsic variation of the BRbS, we analyzed mice from one standard population (six mice from MON population, 2*n* = 40) and mice from one Rb population with a high number of chromosomal fusions (seven Rb mice from VIL population, 2*n* = 31–32) (Fig. 1e, Supplementary Fig. 1f and Supplementary Table 1). We also included three control BL6 mice. The chromosomes were assessed in five categories: (i) acrocentric chromosomes of laboratory mice (Acr BL6), (ii) acrocentric chromosomes of wild-caught standard mice (Acr St), (iii) acrocentric chromosomes of wild Rb mice (Acr Rb), (iv) Rb chromosomes in heterozygous state of wild Rb mice (Met Het), and (v) Rb chromosomes in homozygous state of wild Rb mice (Met Hom) (Fig. 1e). In Rb mice from the VIL population, our analysis indicated that irrespective of the chromosomal complement (2*n* = 31 or 2*n* = 32), the CO distribution per-chromosome arm had the same pattern (Supplementary Fig. 1f). As a result, data from VIL mice were pooled together for subsequent downstream analysis of CO distribution.

When considering the number of COs per-chromosome arm, we detected significant differences between arm types (Acr BL6, Acr St, Acr Rb, Met Het, and Met Hom) (*χ*^2^ test, *P* < 0.05) (Fig. 1e). The frequency of chromosome arms with zero CO was low (≤3%) in standard mice (BL6 mice and wild-caught standard mice), mirroring previous observations^21^. However, in Rb mice with fusions in a homozygous state, we detected a statistically significant increased proportion of chromosomal arms with zero CO (7.45%) (*χ*^2^ test, *P* < 0.05; Fig. 1e and Supplementary Fig. 1f). Moreover, in Rb mice with homozygous fusions, we detected a significant decrease in the frequency of chromosomal arms with two COs (Met Homo, 6.98%) (*χ*^2^ test, *P* < 0.05) when compared with their nonfused counterparts (i.e., acrocentric chromosomes) (Acr Rb, 18.23%) or Rb mice with heterozygous fusions (Met Het, 11.85%) (*χ*^2^ test, *P* < 0.05, Fig. 1e). These observations suggest that the reduced CO frequency observed in Rb mice is due to a reduction of chromosome arms with two COs, especially when the Rb fusions are homozygous (Fig. 1e).

We next examined how chromosomal CO distribution was affected by Rb fusions (Fig. 1f). We found significant differences in CO distribution between standard acrocentrics, Rb acrocentrics, and Rb metacentrics (*χ*^2^ test, *P* < 0.05, Fig. 1f). CO distribution along acrocentric chromosomes (i.e., nonfused) in both standard (MON) and Rb mice (VIL) was distinct in distal regions (90–100% of the chromosome length) (*χ*^2^ test, *P*< 0.05, Supplementary Fig. 1g). In the case of fused chromosomes, single CO events were slightly displaced (although not significant; *χ*^2^ tests, *P* = 0.15) in homozygous fusions toward distal regions (80–90% of the chromosome arm length) when compared to heterozygous fusions (Supplementary Fig. 1g). On chromosome arms with two COs, a bimodal distribution was detected in both standard and Rb mice, with differences between standard acrocentrics, Rb acrocentrics, and Rb metacentrics (*χ*^2^ test, *P* < 0.05, Fig. 1f). In the case of acrocentrics (in both standard and Rb mice), we also detected differences in distal regions (90–100% of the chromosome length) (*χ*^2^ test, *P* < 0.05, Supplementary Fig. 1g). On fused chromosomes in a heterozygous state, the majority of COs were localized either in proximal (from 15 to 55% of the chromosome arm length) or very distal (90–95% of the chromosome length) regions (Supplementary Fig. 1g). For chromosomes in a homozygous state, proximal COs appeared more displaced from the centromere (25–45% of the chromosome arm length). Our results indicate that chromosomal fusions reshape recombination landscapes by reducing both the overall number of COs per-chromosome arm and the distribution of recombination events along chromosomes.

As perturbations in obligatory CO frequencies and overall CO distribution can impair meiosis and affect reproductive fitness, we analyzed sperm viability. Rb mice had a significantly higher fraction of immobile sperm (66.1% ± 21.2 vs. 31.7% ± 8.8, Mann–Whitney test, *P* = 0.006) and increased sperm mortality (72.9% ± 18.5 vs. 32.1% ± 16.2, Mann–Whitney test, *P* = 0.005) when compared with standard mice.

Moreover, and in order to assess the fine-scale genomic impact of both the overall reduction and chromosomal redistribution of COs, we analyzed the landscape of genomic diversity (i.e., number of alleles, observed/expected heterozygosity, and nucleotide diversity, Supplementary Table 2) and divergence (expressed as F_ST_ values, Supplementary Tables 3 and 4) between populations together with estimates of recombination rates based on linkage disequilibrium (expressed as 4N_e_r/kbp) between standard and Rb mice (see “Methods”). Both principal component analysis (PCA) (Supplementary Fig. 2a) and estimations of population structure (Supplementary Fig. 2b) revealed that BRbS mice clustered according to the geographical distribution of populations. Moreover, and consistent with the cytological analysis, we observed an overall reduction of recombination rates (expressed as 4N_e_r/kbp) in Rb mice when compared to standard mice (Mann–Whitney test, *P* < 0.001; Supplementary Fig. 2c, d). Consistent with the variation in recombination rates observed in Rb mice, we also detected differences in molecular diversity between populations (Supplementary Tables 2, 3, and 4). Although estimates of observed and expected heterozygosity were similar between populations, standard mice showed higher allelic richness than in Rb mice (Supplementary Table 2). Concomitant with the overall reduction of recombination rates, Rb mice showed higher F_ST_ estimates than standard mice, both when comparing divergence between standard and Rb populations and between Rb populations (Tukey–Kramer test, *P* < 0.005) (Supplementary Tables 3 and 4).

### Modulation of chromosomal lengths and CO distribution

Variation in both chromatin loop length and chromosomal axis length can alter CO frequencies^17–19^; therefore, we sought to understand how these chromosomal features were affected by chromosomal fusions. We did not observe differences in the total axis length per cell in Rb mice when compared to standard mice (Supplementary Fig. 3a). However, when analyzing chromosomes in Rb mice according to chromosomal arm type, fused chromosome arm length was longer than the acrocentrics (Mann–Whitney test, *P* < 0.0001) (Fig. 2a and Supplementary Fig. 3a). Accordingly, fused chromosomes had significantly lower CO density than acrocentrics (Mann–Whitney test, *P* < 0.0001), irrespective of their state (homozygous or heterozygous) (Fig. 2a). Remarkably, heterozygous fused chromosomes were significantly longer than when in a homozygous state (Wilcoxon test, *P* < 0.0001) (Fig. 2a and Supplementary Fig. 3b). These observations suggest that Rb fusions affect both chromosomal axis length and CO chromosome distribution. Likewise, CO formation is established in a chromosome-specific manner, mirroring previous observations in all-acrocentric mice^17^.

### CO distribution in homozygous and heterozygous fusions

Meiotic DSBs are repaired in the context of the chromosomal axes as homologous chromosomes pair and synapse^27,28^. Thus, disturbances of homologous pairing during prophase I are expected to affect CO patterns. Concomitant with this view, perturbed pairing in Rb mice had an effect on CO distribution (Fig. 2b–e). We analyzed five Rb mice from the VIL population from which material was available, categorizing three different pairing states observed in heterozygous fusions: (i) fully synapsed, (ii) open, and (iii) asynapsed chromosomes (Fig. 2b, c and Supplementary Fig. 3c). Disturbed synapsis influenced CO distribution per-chromosome arm, while the average number of CO events per arm was similar between individuals (Fig. 2c and Supplementary Fig. 3c). That is, while fully synapsed heterozygous fusions generally had one, homogeneously distributed, CO event per arm, asynapsed heterozygous fusions presented COs either in intermediate positions (40–50% of the chromosome length) or in distal regions (80–90% of the chromosome length) (Fig. 2c and Supplementary Fig. 3c). However, synaptic disturbances in heterozygous chromosomes did neither significantly affect CO distribution nor reduce the number of COs per arm when compared to acrocentrics (Fig. 1e), most probably due to variation in chromosomal axis length (Fig. 2a). Interestingly, we observed that 28.57% of the CO events detected in asynapsed chromosomes were located at the border of synapsed and unsynapsed regions (40% of the chromosome length, Supplementary Fig. 3c). These COs could prevent further asynapsis, as previously suggested for hexavalent meiotic configurations in Rb mice^29^.

Both the reduced number of COs per arm and the displacement away from the centromere observed in homozygous fusions compared to heterozygous (Fig. 1e and Supplementary Fig. 1f) were unexpected based on the chromosomal speciation theory^30^, thus, we sought to understand the mechanistic factors behind this pattern. Remarkably, we detected that homozygous chromosomal fusions were associated with the presence of double-centromeric signals at variable frequency among individuals (from 8.33 to 34.45%, Fig. 2d and Supplementary Fig. 3d). We then investigated whether these double-centromeric signals correlated with CO chromosomal distribution by analyzing mice from the VIL population with the same number of fusions (four fusions in a homozygous state and one fusion in a heterozygous state). Notwithstanding cell-to-cell variability, the presence of a double-centromere signal was associated with displacement of COs toward distal regions of the chromosome (Fig. 2d). This extended centromeric recombination suppression, coupled with shorter chromosomal axes, likely explains the significant reduction of COs per arm on homozygous Rb chromosomes when compared to heterozygous (Fig. 1e and Supplementary Fig. 1f).

Overall, we observed that Rb fusions had an effect on CO chromosomal distribution and chromosomal axis length, depending on whether they are in the heterozygous or homozygous state. With consistent genome size, the longer chromosomal axes in heterozygous fusions would be attributed to shorter loops (Fig. 2e). Despite the presence of different levels of asynapsis, heterozygous fusions have longer axes allowing the formation of an obligatory CO per arm necessary for faithful chromosomal segregation. The contrasting pattern found in homozygous fusions (short chromosomal axis, high frequency of arms with zero CO, and centromere interference) suggests that chromosome architecture could play a major role in reshaping CO distribution.

### Chromosomal fusions reorganize spatial chromosome occupancy

We then analyzed whether Rb fusions impact the three-dimensional genome folding in germ cells. Using Fluorescence-Activated Cell Sorting (FACS), we isolated highly enriched meiotic (primary spermatocytes at the pachytene/diplotene stage, P/D) and postmeiotic cells (round spermatids, RS) from wild-caught Rb mice (derived from the same population) with a high number of fusions (Supplementary Table 1, Supplementary Fig. 4a, b and “Methods” section). For each germ cell fraction, we performed in situ Hi-C^3,16^ to generate genome-wide Hi-C maps for primary spermatocytes and round spermatids. The Rb somatic profile was derived from a wild-caught Rb male primary fibroblast cell line belonging to the BRbS. Germ cell and somatic data for all-acrocentric (standard) mice have been published previously^16^. After filtering, an average of 254.9 million valid interactions per cell type was detected (Supplementary Tables 5 and 6) with high correlation values between biological replicates (from 0.96 to 0.92) (Supplementary Fig. 4c and “Methods” section).

**Fig. 3:**
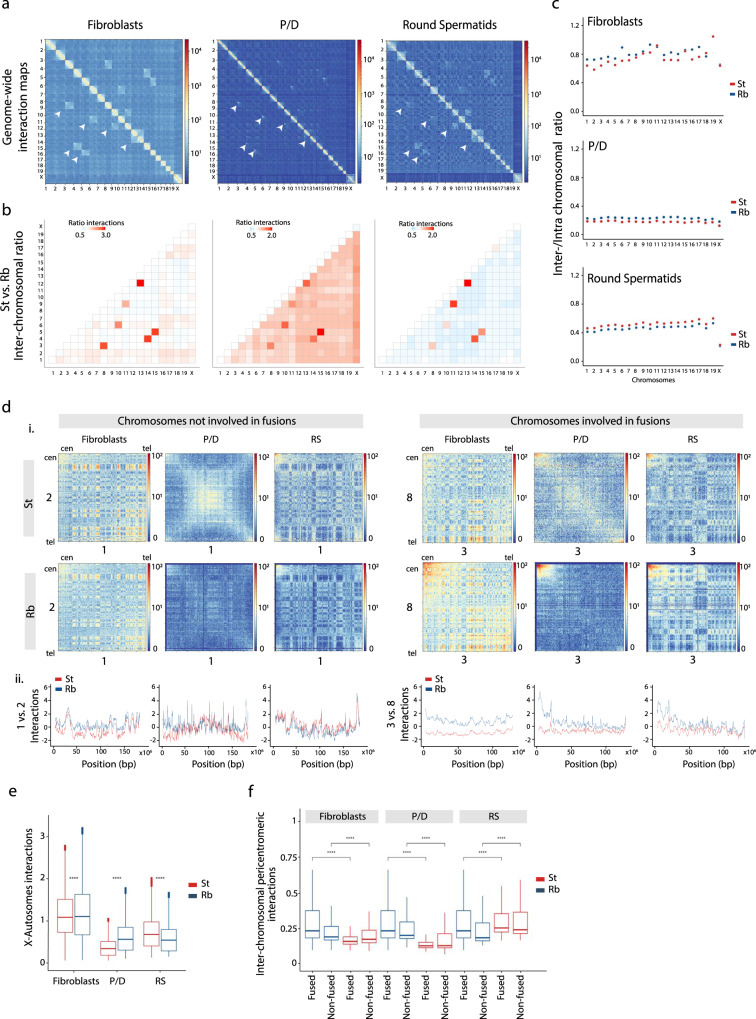
Effect of Rb fusions on the higher-order chromatin structure. **a** Genome-wide ICE-corrected heatmaps (500 kbp) for the cell types analyzed (fibroblasts, pachynema/diplonema—P/D, and round spermatids—RS) in Rb mice. Chromosomes involved in Rb fusions emerge as regions with high interchromosomal interaction in all cell types (arrowheads). Rb fusions are the following: 3.8, 4.14, 5.15, 6.10, 9.11, and 12.13. **b** Heatmaps depicting genome-wide interchromosomal interaction ratio between standard (St) and Rb mice. Chromosomes in red indicate higher interactions in Rb than in standard mice, whereas chromosomes in blue indicate higher interactions in standard than in Rb mice. As expected, all chromosomes involved in fusions show high interaction ratios in all the three cell types analyzed. Source data are provided as a Source Data file. **c** Inter-/intrachromosome interaction ratio for the cell types analyzed (fibroblasts, P/D, and RS) in St and Rb mice. In fibroblasts (upper panel), values are the same for chromosome 19 in both St and Rb mice. Source data are provided as a Source Data file. **d** Examples of interaction patterns between chromosomes involved and not involved in fusions. (i) Interaction heatmaps representing chromosomes 1 and 2 (not involved in fusions) and chromosomes 3 and 8 (fused in a heterozygous state) in both St and Rb mice. In chromosomes 3 and 8, the fusion becomes evident in interaction maps from Rb mice, with high interaction in the pericentromeric region of the chromosomes (0–3 Mbp from the centromere). The observed scaling is consistent across all chromosomes. Source data are provided as a Source Data file. (ii) Interaction plots for fibroblasts, P/D, and RS for chromosomes not involved (1 and 2) and involved in fusions (3 and 8). The observed scaling is consistent across all chromosomes. Source data are provided as a Source Data file. **e** Boxplot showing the number of genome-wide interactions between the X chromosome and autosomes detected in St and Rb mice per each cell type (Mann–Whitney test, ****P < 0.0001, two-sided). Boxplots are presented as mean values (center line) ± SD. Source data are provided as a Source Data file. **f** Boxplots depicting genome-wide interchromosomal interactions per million at pericentromeric regions (from the centromere up to 3.5 Mbp) between St and Rb mice (Mann–Whitney test, ****P < 0.0001, two-sided). Boxplots are presented as mean values (center line) ± SD. For each cell type, two groups of chromosomes were compared: chromosomes involved in Rb fusions (3.8, 4.14, 5.15, 6.10, 9.11, and 12.13) and chromosomes not fused (1, 2, 7, 16, 17, 18, 19, and X). Source data are provided as a Source Data file.

Genome-wide interaction maps confirmed the presence of six Rb fusions (3.8, 4.14, 5.15, 6.10, 9.11, and 12.13) in all cell types analyzed from Rb mice (Fig. 3a and Supplementary Table 1). All chromosomes involved in Rb fusions showed higher interchromosomal interaction values (interaction ratio ~3.0) in Rb mice, compared to standard mice, in all cell types (Fig. 3b and Supplementary Table 1). However, we also detected different patterns of interchromosomal interaction ratios between nonfused chromosomes. In fibroblasts, the few nonfused chromosomes (e.g., chromosomes 1 and 2) had higher values of heterologous interactions in Rb mice when compared to standard mice (Fig. 3b, c and Supplementary Fig. 5). However, the spatial genome architecture was highly reorganized in P/D, affecting nearly all chromosomes (fused and nonfused) (Fig. 3d and Supplementary Fig. 6).

**Fig. 4:**
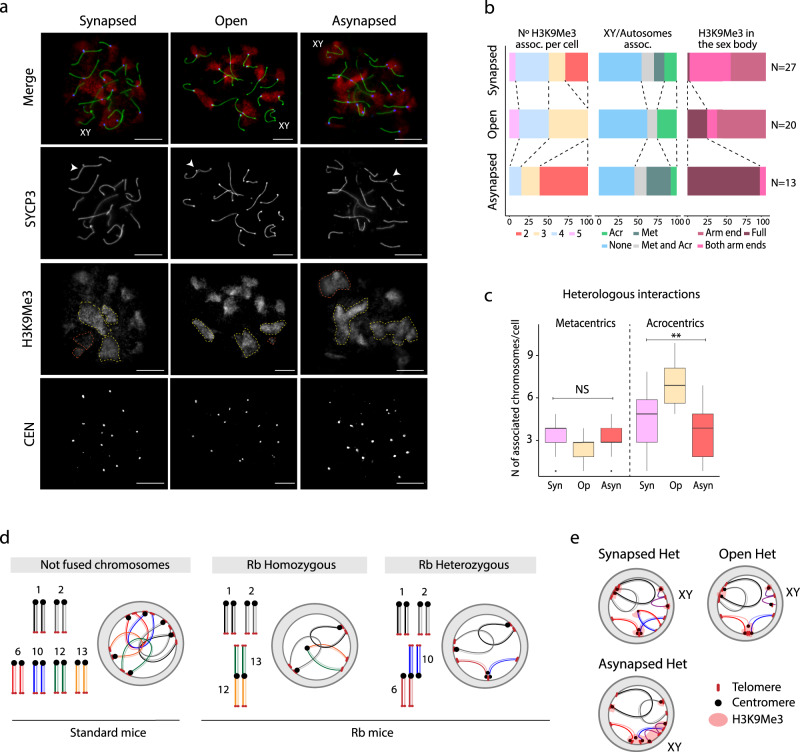
Interchromosomal associations. **a** Examples of immunofluorescence on primary spermatocytes at pachytene stage, labeling the synaptonemal complex with SYCP3 (green), the centromeres (CEN, blue), and H3K9Me3 (red). Immunofluorescence replicates, *n* = 3. SYCP3 staining allowed for the detection of the different heterozygous Rb fusion according to synapsis pattern: synapsed, open, and asynapsed (white arrows). H3K9Me3 shows associations between different chromosomes (yellow dashed lines) and differential distribution in the XY sex body (orange dashed lines): in both arms (as shown in the synapsed example), in one arm end (as shown in the open example), or fully around the sex body (as shown in the asynapsed example). The sex body is indicated as XY. Scale bar = 10 µm. **b** Analysis of H3K9Me3 associations according to synapsis states (synapsed, open, and asynapsed) of heterozygous metacentrics (left panel), sex chromosome/autosome associations (central panel), and the sex body on its own (right panel). Acr acrocentric chromosomes, Met metacentric chromosomes, assoc. association. *N* = number of cells analyzed. Source data are provided as a Source Data file. **c** Number of associated chromosomes (metacentrics (*n* = 60)) or acrocentrics (*n* = 60)) detected per cell depending on the synapsis state of heterozygous metacentrics (Kruskal–Wallis, **P ≤ 0.005, one-sided). Boxplots are presented as mean values (center line) ± SD. syn synapsed, op open, asyn asynapsed, ns nonsignificant. Source data are provided as a Source Data file. **d** Schematic representation of chromosome organization in P/D according to the presence of Rb fusions. In standard mice, all chromosomes are acrocentric and are attached to the nuclear lamina. When Rb fusions are present, chromosome organization is disrupted, affecting chromosome disposition inside the nucleus, either in a homozygous or a heterozygous state. **e** Schematic representation of the centromeric associations detected with the H3K9Me3 signal in addition to the XY disposition according to synapsis pattern of heterozygous Rb fusions (i.e., synapsed, asynapsed, or open). het heterozygous metacentric.

In P/D, all chromosomes showed high values of heterologous interactions in Rb mice (interaction ratio ≥1.5) for all chromosomes when compared to standard mice (Fig. 3b and Supplementary Fig. 5), suggesting a genome-wide redistribution of the spatial disposition of chromosomes inside nuclei. The presence of heterologous associations was further demonstrated by the immunodetection of the centromeric constitutive heterochromatin (exemplified as H3K9Me3 signals) on pachytene chromosomes of Rb mice (Fig. 4a). In fact, heterologous interactions were dependent on the pairing state of Rb fusions (i.e., fully synapsed, open, or asynapsed) (Fig. 4b). Specifically, we observed more centromeric associations of acrocentric chromosomes when Rb fusions showed open configurations (Fig. 4c). In addition, the sex body (the X and the Y chromosome) was generally isolated from autosomes (Fig. 4b). When heterozygous Rb fusions failed to fully synapse, there was a general disruption of interchromosome associations, with an increased number of chromosomal associations (Fig. 4c). Moreover, the sex body showed abnormal signals of heterochromatization being frequently associated with fused chromosomes (Fig. 4b). Interestingly, we detected full heterochromatinization of the sex body in >90% of cells with asynapsed heterozygous Rb fusions (Fig. 4b), which could contribute to the increased X/autosome heterologous interactions in P/D Rb mice (Fig. 3e). In contrast, heterologous interactions were reduced in RS for all chromosomes not involved in fusions (ratios < 1, Fig. 3b and Supplementary Fig. 5), a pattern confirmed by the analysis of inter-/intrachromosomal interactions (Fig. 3c). In fibroblasts and P/D, Rb mice showed higher inter-/intrachromosomal interaction ratios than all-acrocentric mice (Mann–Whitney test, *P* < 2.2e-16), a pattern that was clearly reversed in RS with higher interaction ratios in standard mice than in Rb mice for all chromosomes (Mann–Whitney test, *P* < 2.2e-16) (Fig. 3c).

Chromosome-specific interaction maps also revealed altered three-dimensional chromosome folding in Rb mice, especially in prophase I (Fig. 3d). For nonfused chromosomes (e.g., chromosomes 1 and 2, which represented a pattern consistent across nonfused chromosomes), interaction patterns were disrupted during P/D in Rb mice (Fig. 3d and Supplementary Fig. 6), losing the interchromosome interaction pattern previously described for standard mice^14,16^. These results suggest the presence of genome-wide conformational changes triggered by the presence of Rb fusions, which was supported by the cytological analysis of chromosome associations (Fig. 4c). For fused chromosomes (e.g., chromosomes 3 and 8, which have a pattern consistent across fused chromosomes), we not only detected changes in mid-chromosome interactions, but also high interaction values in pericentromeric regions (Fig. 3d–f and Supplementary Fig. 6).

Overall, our data suggest that the presence of Rb fusions reorganize chromosomal nuclear occupancy genome-wide (Fig. 4d, e), increasing the rate of heterologous interactions in primary spermatocytes. Moreover, our results suggest that pairing disturbances of heterozygous fusions during prophase I have a direct impact in chromosome spatial distribution (Fig. 4e). As such, when heterozygous fusions are synapsed, more permissiveness for centromeric associations between acrocentric chromosomes is observed and the sex body is generally isolated at the periphery of nuclei. Conversely, when heterozygous Rb fusions fail to fully synapse, there is a general disruption of interchromosome associations and the sex body shows abnormal signals of heterochromatinization and frequently associates with fused chromosomes (Fig. 4e).

### Higher-order chromatin remodeling

**Fig. 5:**
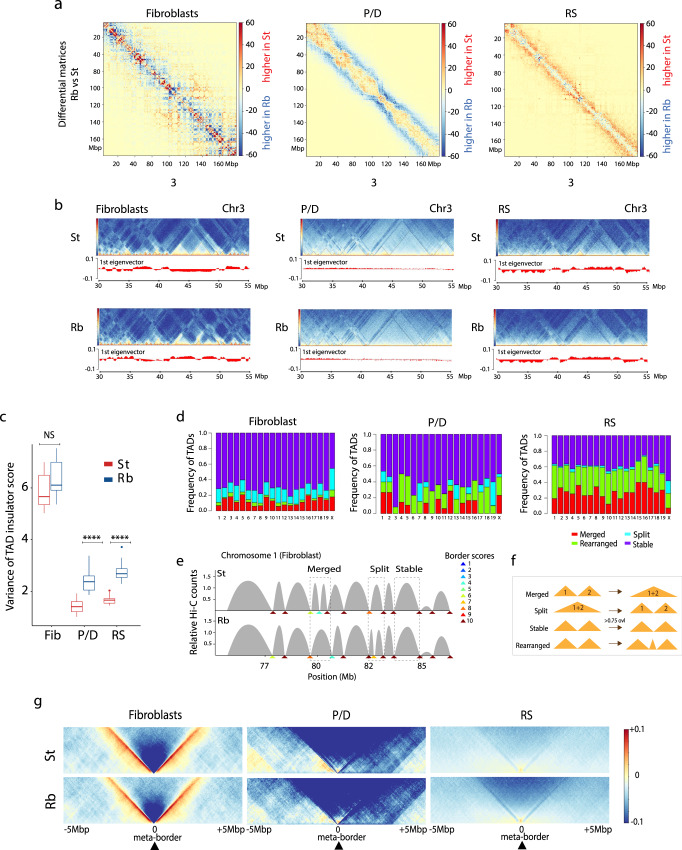
Variance in fine-scale compartmentalization. **a** Differential Hi-C matrices (log_2_ of fold change using Rb mice as a reference when compared with standards) for chromosome 3 in cell types analyzed (fibroblasts, pachynema/diplonema—P/D, and round spermatids—RS), at a 500-kbp resolution. The observed scaling is consistent across all chromosomes. Red indicates higher number of interactions in standard (St) mice when compared to Rb mice, whereas blue represents higher number of interactions in Rb mice. **b** Chromosome (Chr) 3 region-specific ICE-corrected heatmaps at 50 kbp (from 30 to 55 Mbp), depicting compartment signal (1st eigenvector) for all cell types. Source data are provided as a Source Data file. **c** Variance of TAD insulator score between St and Rb mice in all cell types (Mann–Whitney test, ****P < = 0.0001, ns *P* > 0.05, two-sided). Boxplots are presented as mean values (center line) ± SD. ns nonsignificant. **d** Frequency of TAD reorganizations between standard and Rb for fibroblasts, P/D, and RS. Source data are provided as a Source Data file. **e** Example of TAD border alignments along chromosome 1 of fibroblasts (from 75 to 86 Mbp). Examples of merged, split, and stable TADs are indicated. TAD border scores are also shown, informing of the TAD boundary strength. Source data are provided as a Source Data file. **f** Schematic representation of TAD reorganization. Merged TADs are the result of fusing two different TADs. Split TADs are those in which one TAD is divided into two TADs. TADs are considered stable when there is an overlap above 75%. When TADs are found in a different organizations, they are considered rearranged. **g** Metaplots for all TAD boundaries detected in Rb mice: fibroblasts (*n* = 2378), P/D (*n* = 288), and RS (*n* = 3798). Data on standard mice were extracted from Vara and colleagues^16^.

We further investigated whether the presence of Rb fusions had an effect on chromatin remodeling at a finer scale (e.g., compartments). In somatic cells, the genome-wide analysis of differential Hi-C matrices showed higher interactions at shorter genomic distances in standard mice than in Rb mice (Fig. 5a). This interaction pattern, which is dependent on genomic distance, was switched at genomic distances of ~6 Mbp in fibroblasts. This was concomitant with slight changes in A/B compartments (Fig. 5b), with Rb mice showing a smaller mean compartment size (0.85 Mbp) than standard mice (1 Mbp).

Mirroring previous studies^14,16^, our genome-wide analysis showed that most compartments were mostly lost in primary spermatocytes (P/D) in Rb mice (Fig. 5b), when homologous chromosomes condense, align, pair, synapse, and recombine. Consistent with this absence of compartments during prophase I, eigenvector values were close to 0 (Fig. 5b) and inter-/intrachromosomal interaction ratios reached a minimum for all chromosomes (Fig. 3c). Importantly, the analysis of differential Hi-C matrices and intrachromosomal interaction ratios revealed that, on average, probabilities of interactions were higher in Rb mice at genomic distances larger than 10 Mbp. Local adjustments in chromatin packing density due to differences in loop positioning and size, together with variations in chromosomal axis length (as revealed by our cytological observations), likely give rise to the differences between standard and Rb mice Hi-C maps.

Robertsonian mice showed a distinctive interaction pattern in postmeiotic cells, with a high incidence of interactions at shorter genomic distances (≤5 Mbp) (Fig. 5a). The effect of highly condensed chromatin in RS of Rb mice would increase short-range contacts (relative to long-range contacts), as we observe. Although A/B compartments reappeared in RS, they were present as a blurry plaid pattern of larger mean size (0.93 Mbp in Rb mice) than in fibroblasts. Interestingly, the proportion of genomic bins with the same compartment status (A or B) was higher between standard and Rb mice in RS (95% bins conserved, *r*^2^ = 0.94) than in fibroblasts (88.24% bins conserved, *r*^2^ = 0.80) (Fig. 5b). These results, together with the low inter-/intrachromosomal interaction values detected in Rb mice (Fig. 3c), suggest that Rb fusions induce physical constraints in RS most likely due to the spatial organization of chromosomes around the chromocenter^31,32^, thus favoring local interactions.

### Chromosomal fusions induce TAD reorganization

Chromosome fusions also affected chromatin remodeling at the sub-megabase scale as revealed by the number of TADs detected and the robustness of their boundaries at a 50-kbp resolution (Fig. 5c–f and Supplementary Fig. 7). Similar to previous observations in standard somatic cells^16^, TADs were well defined in Rb fibroblasts (Fig. 5c–f), with a total of 2391 detected. With an average length of 1.14 Mbps, TADs in Rb fibroblasts had a higher variance of insulation scores than in standard fibroblasts (Fig. 5c). Nevertheless, the majority (70%) of TADs were stable (Fig. 5d), with general preservation of border conformation, as revealed by the TAD meta-border plot (Fig. 5e) that shows the specific border interactions of loop domain TADs^3,33^.

In primary spermatocytes, there was a substantial reduction in the variance of TAD insulation score, and reduction of total TADs detected, when compared to somatic cells (*n* = 288 in Rb and *n* = 294 in standard). The TAD insulation score variance was higher in Rb mice (Fig. 5c). Moreover, meta-border plots show asymmetric TAD borders (Fig. 5g), more characteristic of stripe TAD domains^33^. The variance of TAD insulation scores was partially recovered in RS, being higher in Rb mice than standard. In RS, Rb mice had fewer but larger (*n* = 3805, 0.71-Mbp average length) TADs than standards (*n* = 4649, 0.59-Mbp average length). Only 40% of TADs were stable in RS (Fig. 5d). The presence of weaker TAD borders in both standard and Rb RS (mean TAD insulator score = 5.46), when compared to fibroblasts (mean TAD insulator score = 8.8), could explain the difference in TADs observed between these cell types, with TADs more prone to reorganize in RS (Fig. 5d). In fact, rearranged, split, and merged TADs showed significantly lower TAD border scores than stable TADs (Mann–Whitney test, *P* = 2e-14). Meta-border plots showed a blurred pattern, consistent with loop domain TAD borders and weak TAD insulator scores (Fig. 5g).

### Olfactory receptor genes and interchromosomal interactions

**Fig. 6:**
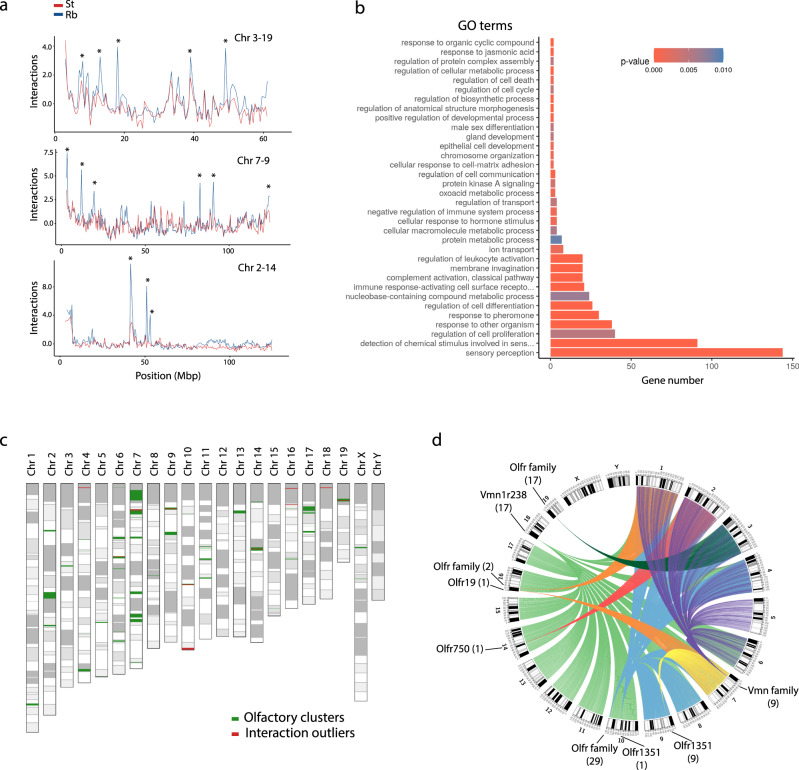
Interchromosomal interaction and olfactory receptors. **a** Interaction profiles of pairs of chromosomes (3 and 19, 7 and 9, and 2 and 14) in primary spermatocytes (pachynema/diplonema, P/D) of Rb mice. In all three examples, interchromosomal interactions are depicted by asterisks. St standard mice, Rb Rb mice, chr chromosome. Source data are provided as a Source Data file. **b** Gene Ontology Enrichment Analysis (GOEA) of genes in newly detected interchromosomal interactions in pachynema/diplonema of Rb mice. Only significant gene ontology (GO) terms with an adjusted *P* value below 0.01 are shown. The *X* axis represents the number of genes associated to each GO term. Source data are provided as a Source Data file. **c** Mouse ideogram showing the localization of olfactory clusters (green) described in literature^34^, and the interchromosomal interactions detected in this work (red). **d** Circus plot representing the interchromosomal interactions in the mouse genome related with sensory perception, which are mostly genes from the olfactory (Olfr) and vomeronasal (Vmn) receptor family. The number of genes found in each region and the gene family are shown in parenthesis. Source data are provided as a Source Data file.

The increased rate of heterologous interactions detected in Rb primary spermatocytes resulted in the emergence of new interchromosomal interactions (*n* = 249, representing 0.5% of the mouse genome) that involved all chromosomes (e.g., chromosomes 2, 3, and 7, Fig. 6a). To explore the potential role of Rb-specific interchromosomal interactions, we analyzed the gene ontology of the 2000 genes contained in these regions. Importantly, we detected enrichment for sensory perception genes (Fig. 6b), specifically, olfactory receptors (OR) (*n* = 118) and vomeronasal receptors (VR) (*n* = 92). These OR/VR genes with heterologous interaction were located in 11 out of the 94 OR clusters previously described in the mouse genome^34^ (Fig. 6c). These included mouse chromosomes 4, 6, 7, 9, 10, 14, 16, 18, and 19. Interestingly, we detected that individual OR clusters interacted with a wide range of regions from different chromosomes, with interactions being either chromosome-specific (e.g., the cluster on chromosome 19 interacts solely with chromosome 3) or multichromosomal (e.g., interactions between two clusters on chromosome 10 with multiple regions of chromosomes 1, 2, 3, 4, 7, 8, and 9) (Fig. 6d). Furthermore, we detected significantly more repeats in OR gene clusters when compared with the Rb-specific interchromosomal interactions and with the rest of the mouse genome (Supplementary Fig. 8a) (Wilcoxon test, *P* ≤ 0.0001), particularly LINE/L1, and LTR/ERV family retrotransposons (Supplementary Fig. 8b).

## Discussion

Here, we provide evidence that chromosomal fusions affect three-dimensional genome topology and meiotic recombination, highlighting the implications of large-scale genome reorganizations on genome function and fertility. Our results show that chromosomal fusions pose important mechanistic constraints in the nuclear architecture of germ cells, affecting heterologous interactions, chromosomal synapsis, and meiotic recombination. This was reflected at different hierarchical levels: (i) chromosomal nuclear occupancy, (ii) inter- and intrachromosomal interactions, (iii) chromosomal axis length, and (iv) topological domains.

Our high-throughput analyses in combination with cytological observations show that disturbances in nuclear chromosomal occupancy occur genome-wide, affecting not only chromosomes involved in Rb fusions but also unfused chromosomes. The pattern observed in primary spermatocytes is especially relevant as fundamental cellular processes take place, such as synapsis and pairing of homologous chromosomes and the formation and repair of DSBs. It is also in primary spermatocytes where DNA loops are organized along the chromosomal axis, with loop size and axis length inversely correlated^17,18^, which can covary within gametes^19^. We detected that the presence of Rb fusions can disrupt the standard nuclear architecture of peripherally attached acrocentrics to center-localized metacentrics^35^, ultimately inducing ectopic heterologous interactions. Such alteration in nuclear occupancy genome-wide, together with the pairing disturbances observed with fusions in a heterozygous state, detected in a chromosome-specific manner, would trigger stochastic physical interactions and expose chromosomal domains to novel regulatory environments. Remarkably, this disruptive pattern also affected chromosome axis length and, as a result, higher-order chromatin remodeling. As chromatin is organized into DNA loops that emerge out of meiotic chromosomal axes^36^, variations in axis length alter the number and size of these loops^19^, ultimately affecting medium- and long-range interactions. Given that the assembly of chromatin loops into chromosomal axes, and the formation and repair of meiotic DSBs (and subsequent COs) are interconnected^18,19,37^, the remodeling of chromosomal interactions translated into a reorganized recombination landscape in Rb mice. Thus, Rb fusions pose mechanical constraints on the spatial genome architecture that affect not only the hierarchical three-dimensional organization of the genome, but also the chromosomal distribution of CO events. The molecular mechanisms behind this phenomenon remain unknown.

Importantly, the redistribution of COs across chromosomal arms was consistent with the observation of low recombination rates at a fine scale (kbp), and high genetic divergence genome-wide. This provides evidence that disturbances in CO distribution due to chromosome fusions result in detectable genomic footprints. According to the ‘suppressed recombination’ model^20^, a reduction in recombination is expected within reorganized regions in the heterokaryotype (e.g., heterozygous Rb fusions). Although this was consistent with our observations (the proportion of chromosome arms with zero CO was slightly higher in heterozygous Rb fusions than nonfused chromosomes), we also detected a strong reduction of recombination in homozygous Rb-fused chromosomes, mirroring early cytological observations^38^. Decreased recombination in homozygous Rb fusions is counterintuitive but could be explained by the presence of double-centromeric signals that represented misaligned centromeres^39^. Since centromeres can reduce recombination rates (so-called centromere interference^40^), the presence of double centromeres could magnify this effect, interfering with both the formation of COs during prophase and subsequently affecting chromosome segregation in metaphase I. Previous studies in BRbS reported disturbances in heterochromatization at the centromeres of fused chromosomes^21^, and a higher frequency of apoptotic spermatogenetic cells in homozygous mice than in heterozygous mice^41^. In fact, the presence of Rb fusions can induce subfertility, as revealed by our observations of the significant reduction in sperm motility and vitality between Rb and standard mice. This, together with previous reports on the variation of the sperm head morphology and spermatogenic activity in Rb mice^42^, suggests that Rb fusions have an effect on mouse fertility, although mild enough to allow their persistence within populations.

Remarkably, we also detected that chromosomal axes of Rb fusions in the heterozygous state were longer when compared to nonfused chromosomes. Considering that the presence of Rb fusions can alter the nuclear disposition of chromosomes during prophase I, this can lead to a delay in synapsis in heterozygous Rb fusions, resulting in the detection of elongated chromosomal axes in a chromosome-specific manner. Given that chromosomal axes elongate, the substrate for the formation of COs increases in heterozygous Rb fusions, as both features are correlated^18,19^. Conversely, the cytological data indicated the presence of shorter chromosomal axes in homozygous Rb fusions (and therefore, longer DNA loops). This was consistent with the Hi-C data, suggesting that Rb fusions induce variance in higher-order chromatin organization. This adds to initial reports on global modulation of chromosomal CO frequencies by both chromatin loop size and chromosomal axis length^17–19^, further suggesting that recombination landscapes can be altered within cells by Rb fusions affecting both the loop/axes length ratio and the spatial chromosome occupancy.

Likewise, Rb fusions not only pose restrictions for chromosomal interactions in primary spermatocytes but also in postmeiotic cells. Chromosomes are highly condensed in round spermatids with all centromeres associated in the center of the cell forming the chromocenter^16^. As heterologous contacts were reduced in Rb mice, we hypothesize that chromosomal fusions restrict interactions between nonfused chromosomes in round spermatids. This restricted and condensed pattern favors intrachromosomal contacts, resulting in the reorganization of TADs in postmeiotic cells.

Our observations have important evolutionary and developmental implications. The dynamic three-dimensional genome topology of germ cells can be affected by chromosomal fusions in two ways: (i) altering chromosomal nuclear occupancy, and (i) reshaping landscapes of recombination. The redistribution of chromosomal nuclear occupancy in spermatocytes that result from Rb fusions brings new genomic regions into close proximity, predisposing to the occurrence of additional rearrangements, as previously suggested^43,44^. Moreover, rearranged nuclear occupancy would expose chromosomal domains to novel regulatory environments, potentially affecting gene expression and/or regulation, as initially proposed by the integrative breakage model of genome architecture^5,6^. As such, new chromosomal interactions resulting from chromosomal fusions may rewire or attenuate gene networks, providing new grounds for evolutionary novelty in the long run. This was the case for olfactory receptor family clusters detected in meiotic-specific interchromosomal interactions in Rb mice. As this gene family is expressed in the male germ line^16^, with a function in spermatogenesis and fertilization^42^, altered regulation of their expression could have an adaptive role. In fact, interchromosomal interactions between OR genes has been previously described in sensory neurons^45^, suggesting that associations between multiple chromosomes can selectively regulate transcription of individual OR genes. Further functional studies will be needed to support this hypothesis in the germ line.

Here, we demonstrate that chromosomal fusions affect the three-dimensional genome-wide topology in germ cells, ultimately reshaping recombination landscapes. The modulation of recombination implies a close interplay between different factors that are involved in chromatin remodeling, centromere interference, and chromosomal axis length. Documenting how such changes in genome organization affect gene expression and regulation is an important dimension for further understanding the effect of genome reshuffling on evolution and fertility. Our results can provide the impetus for the exploration of the functional and structural basis of genomes in a broad context, reinforcing the link between the three-dimensional genome architecture, genome integrity, and fertility.

## Methods

### Animals and cell lines

We sampled a total of 63 wild-caught house mice (*M. m. domesticus)* from six populations, covering the extent of the BRbS (Fig. 1a and Supplementary Table 1). All animals included in the study were previously karyotyped^21,23,46^ confirming the presence of Rb fusions (Supplementary Table 1). Three males from the laboratory strain BL6 were also included in the recombination analysis. Animals were housed and treated in strict accordance with ethical guidelines approved by the Universitat Autònoma de Barcelona (Spain).

The BRbS system mice included in the present study consisted of three populations without Rb fusions (2*n* = 40; MON, BOI, and Olost) and three populations with Rb fusions (2*n* = 39–28; SS, CAS, and VIL). Mice from Rb populations are characterized by having between one and six Rb fusions involving 12 different chromosomes (Rb(3.8), Rb(4.14), Rb(5.15), Rb(6.10), Rb(9.11), and Rb(12.13)), either in heterozygous or homozygous states (Supplementary Table 1)^23,24^. The BRbS is characterized by Rb fusions present as chromosomal polymorphisms, thus not fixed within populations.

Moreover, a primary fibroblast cell line derived from a male mouse from the BRbS system (#954, 2*n* = 30) previously established in our lab^46^ was used as a somatic control in the Hi-C experiments. Cells were cultured in DMEM medium supplemented with 10% fetal bovine serum and 1% PenStrep at 37 °C and 5% CO_2_.

### Spermatocyte spreads and immunofluorescence

Direct analyses of recombination are normally based on the detection of either COs or their final products, the chiasmata, visible cytogenetically in meiocytes in later stages of the first meiotic division (i.e., pachytene chromosomes)^18,37^. Here, we analyzed the physical location of COs along the axes of pachytene chromosomes using the immunofluorescence staining technique to detect MLH1, a protein that localizes type I (interfering) COs along with one of the proteins involved in synaptonemal complex (SC) formation (the synaptonemal complex protein 3, SYCP3). The chromosomal distribution of MLH1 foci can be considered a proxy for the positioning of COs^18,47,48^. The position of the centromeres along the chromosomal axes was visualized by staining centromeric proteins using the sclerodactyly and telangiectasia (CREST) serum (Fig. 1a and Supplementary Table 1). In addition, we also detected RAD51 (marker of programmed DSBs) at the early stages of prophase I and H3K9Me3 (a marker of centromeric constitutive heterochromatin). Spermatocyte spreading and immunofluorescence were performed^48^. Testes were mechanically disaggregated until obtaining a cell suspension in 1× PBS. The cell suspension was then distributed into different slides and incubated with 1% lypsol for 16 min followed by a 20-min incubation with 4% paraformaldehyde. Then slides were left to dry and washed twice with PhotoFlo 1% (Kodak) and then blocked with PBS–Tween-20 (0.05%). Slides were incubated overnight at 4 °C with the following primary antibodies: anti-mouse MLH1 (BD Pharmingen Cat# 551092, 1:50), anti-human CREST (courtesy of M. Fritzler, 1:100); anti-rabbit SYCP3 (Abcam Cat# ab15093, 1:400), anti-rabbit H3K9me3 (Abcam Cat# ab8898, 1:300), and anti-mouse RAD51 (Millipore Cat# PC-130, 1:100). Primary antibodies were detected with anti-rabbit Cy3 (Jackson ImmunoResearch Laboratories Cat# 111-165-003, 1:200) combined with anti-mouse FITC (Jackson ImmunoResearch Laboratories Cat# 115-095-003, 1:200) and anti-mouse Cy5 (Jackson ImmunoResearch Laboratories Cat# 115-175-146, 1:200). Spermatocyte preparations were visualized and captured using a Zeiss Axioskop epifluorescence microscope equipped with the appropriate filters and a charged coupled device camera (ProgRes^®^ CS10plus, Jenoptik).

### Sperm analysis

Analyses were conducted in a subset of 15 of male mice from the BRbS. This included six standard mice from BOI population and nine mice with Rb fusions from VIL and CAS populations. Briefly, the right epididymis was obtained for each specimen, and the caudal portion was drained to acquire epididymal spermatozoa. The process was monitored with an Olympus SZ30 stereoscope microscope. Spermatozoa were processed following the protocols for the examination and processing of human semen samples described by the World Health Organization^49^. Around 1000 spermatozoa were analyzed per individual using an Olympus CH30 microscope.

For each sample, a slide with a drop of fresh spermatozoa suspension was analyzed. Depending on the characteristics of the spermatozoa movement, they were classified into the following categories: (i) fast progressive and linear motility, (ii) slow progressive motility by swinging or doing circular movements, (iii) nonprogressive motility but the movement of the head/tail, and (iv) no motility. In order to determine sperm vitality, a drop of eosin Y (0.5%, Sigma-Aldrich) and a drop of sperm suspension were mixed on a slide. After 2 min, the preparation was assessed under the microscope. This method allows the identification of live (not stained) or dead spermatozoa (stained by the inability to expel eosin).

### CO data analysis

For the analysis of COs and centromere position, only pachytene spermatocytes were considered. Only axis-associated MLH1 foci were counted considering the number of MLH1 foci per arm, chromosome, and per cell. SC length (expressed in μm) was calculated as the mean length of all autosomal SCs per cell in individual mice. Thus, we measured the SC length of each chromosome and the physical distance between COs. For the analysis of RAD51, only leptotene and zygotene spermatocytes were considered.

The Micromeasure 3.3 software^50^ was used for the analysis of chromosome-specific recombination maps based on the distances between adjacent MLH1 foci^18,48^. For each chromosome, the position of each MLH1 focus was recorded as a relative position (percentage of the SC total length), identified by the immunofluorescence signal of the centromeres in each cell. Discrimination of heterozygous chromosome states was accounted for, in parallel with the presence of double-centromere signals in homozygous metacentrics. Detailed analyses of CO chromosomal distribution, asynapsis, and double centromeres, were conducted in animals belonging to one standard population (MON) and one Rb population (VIL).

CO frequency plots were constructed for each chromosome type (acrocentric, Rb in heterozygous state, and Rb in homozygous state). As CO data were not normally distributed, analysis of variation in the number and position of MLH1 foci along chromosomes among different groups was assessed using nonparametric tests, including Bartlett’s test (*P* = 0.1372), thus demonstrating equal variance. Accordingly, the Mann–Whitney test was used in comparisons between groups, Kruskal–Wallis to test intragroup differences (either by population or type of fusion), and Wilcoxon’s rank-sum test followed by Dunn’s tests adjusted by Bonferroni for multiple comparisons. Data were expressed as the mean ± standard deviation (SD). In addition, Pearson’s *χ*^2^ test was used to compare arm proportions of the different number of MLH1 foci per arm type and the differences in the relative distribution of MLH1 foci along chromosomal arms and between chromosomal arms according to the presence of Rb fusions (acrocentric nonfused, heterozygous metacentric, and homozygous metacentric). Moreover, Spearman correlations were calculated to test relationships among MLH1 and RAD51 foci, MLH1 foci and diploid number, and MLH1 foci and the number and state of chromosomal fusion. Spearman correlation analysis was also performed for MLH1 foci and chromosome arm length. In all statistical analyses, a *P* ≤ 0.05 was considered statistically significant.

### SNP genotyping

Analyses of genomic divergence and recombination rate at the Mbp scale were conducted using genotyping data from a subset of 34 mice from two standard populations (2*n* = 40; BOI and Olost) and two Rb populations (2*n* = 28–39; SS and CAS populations) retrieved from^24^ (Supplementary Table 1). Data consisted of the Mouse Universal Genotyping Array (MegaMUGA), which consisted of 77,808 evenly distributed SNP markers built on the Illumina Infinium platform^51^. SNPs were filtered to remove markers with missing values >5% threshold using PLINK version 1.9^52^. This resulted in a final data set of 63,344 informative SNPs distributed across all chromosomes, with the exception of chromosomes 8 and Y. The final data set was considered for subsequent analyses of genome-wide screening of divergence and estimates of recombination rate.

The ADMIXTURE software^53^ was used to estimate individual ancestry and admixture proportions assuming K populations based on a maximum likelihood method. Analyses were run only for SNPs with a greater than 95% genotype call. The numbers of clusters (K) were evaluated applying Evanno’s ΔK^54^ with three different *K* values (*K* = 3, 4, 5) showing the lowest likelihood values. ADMIXTURE analyses were plotted using the R package Pophelper v2.3.0^55^. In addition, multiple dimensional scaling analysis was performed using PLINK, by first generating a genome file (–genome flag) from the vcf file containing the SNPs, and then the mds file that was plotted in the R environment.

### Genome-wide screening of genetic divergence and diversity

We estimated the number of alleles (Na), allelic richness (A_r_), observed heterozygosity (Ho), expected heterozygosity (H_e_), inbreeding coefficient (F_IS_), and nucleotide diversity (pi). Na and Ar were estimated using the hierfstat package v0.04-22^56^ implemented in R. Allelic richness was refracted for a minimum of 22 alleles (or 11 diploid samples), which was the lowest observed sample size between the three groups. Ho and H_e_ were calculated using PLINK v1.90b6.12^52^, and F_IS_ and pi with VCFtools 162 v.0.1.16. One-thousand bootstraps were performed for pairwise F_ST_^57^ estimations with the StAMPP v1.6.1 package in R^58^.

Pairwise F_ST_ comparisons were conducted between populations genome-wide and considering chromosomes involved in fusions (3, 4, 5, 6, 8, 9, 10, 11, 12, 13, 14, and 15) and not involved in fusions (1, 2, 7, 16, 17, 18, 19, and X). Estimated F_ST_ values were adjusted with the Bonferroni correction to minimize type I errors. Tukey–Kramer tests (JMP package version 5.1.2, SAS Institute Inc.^59^) were used to analyze differences between groups.

### Estimates of recombination rates

The program LDhelmet^60^ was applied for the estimation of recombination rates at a fine scale (kbp). As LDhelmet has a 25-diploid-sample limit (50 haplotypes), we sampled a random subset of 25 individuals from the 34 individuals included in the SNP analysis, using the vcftools —max-indv option and by chromosome using —recode and —chr^61^. LDhelmet estimates the recombination rates from phased chromosomes or haplotypes from a population; thus, we phased our data using the software SHAPEIT^62^ by using the option –rho 0.001. Once phased, each chromosome vcf file was split into two groups, according to standard or Rb samples. Each file was then transformed to LDhelmet input snps and pos files with the —ldhelmet flag. The likelihood tables were generated using LDpop^63^, and then transformed to LDhelmet format following the software’s manual indications. We performed the analysis per-chromosome, based on the SNP data. Estimations of the population-scaled recombination rate ρ = 4*N*_e_*r* were obtained using the parameters recommended by software’s developers, where *N*_e_ is the effective population size and *r* the genetic map distance across the region analyzed. Using this approach, we established recombination rates in windows of 50 SNPs across the mouse genome considering two groups: standard and Rb mice. Mann–Whitney tests were used to analyze differences between groups.

### FACS of mouse male germ cells

Testis cell disaggregation and FACS were conducted^16^. Briefly, germ cells at a concentration of 1 million per 500 µl were incubated in formaldehyde (1%) for 10 min prior to FACS. Glycine (0.125 M) was added and incubated with agitation at room temperature for 5 min and then at 4 °C for 15 min. Cells were then centrifuged for 10 min at 290 × *g* at 4 °C and resuspended in 3 ml of 1× PBS with Hoechst staining.

Germ cells were sorted using a BD Influx^TM^ (BD Biosciences) coupled with the BD FACS^TM^ software (version 1.0) and an ultraviolet laser (355 nm). Subsequently, two main germ cell populations (P/D and RS) were isolated by plotting Hoechst Blue (UV355–460/50) vs. Hoechst red (UV355–670/30) emissions to discriminate cells by both their DNA content and their complexity. Cell populations were collected after sorting in 1× PBS and centrifuged for 5 min at 1800 × *g*. The supernatant was discarded, and cell pellets were flash-frozen at −80 °C until use. Sorting experiments lasted between 3 and 6 h to collect between 0.2 × 10^6^ and 3.2 × 10^6^ cells, depending on the germ cell population.

Cell enrichment of each flow-sorted population was evaluated by immunofluorescence using specific meiotic proteins and DAPI morphology. For primary spermatocytes, prophase-I stages (leptonema, zygonema, pachynema, and diplonema) were identified based on SYCP3 (1:400) and ɣH2AX patterns (1:300). Cell enrichment of round spermatids was determined based on nucleus morphology and DAPI pattern^16^. Cells were fixed on slides and then mounted with DAPI diluted in Vectashield (Vector Laboratories). Slides were analyzed using fluorescence microscopy (Axiophot, Zeiss) coupled with a ProgRes*®* CS10plus, Jenoptik camera. Representative images were captured with ACO XY (A. Coloma, Open Microscopy). Between 50 and 100, cells were counted for each flow-sorted population. Only sorted populations with an enrichment above 80% were considered for subsequent experiments.

### In nuclei Hi-C

The generation of Hi-C libraries was conducted following Vara and collaborators^16^. Rb mice included in the Hi-C analysis were selected based on their karyotype characteristics (high number of Rb fusions) and availability of testis material. All mice included in the Hi-C experiments belong to the same population (VIL) and were included in the recombination analysis, showing similar patterns of CO distribution (Supplementary Fig. 1f and Supplementary Table 1). Two replicates for cell type were obtained from a total of 3.4 × 10^6^ primary spermatocytes at the P/D stage, 12.8 × 10^6^ round spermatids previously isolated by FACs. In addition, a total of 10 × 10^6^ Rb fibroblasts (two biological replicates) was also included. Libraries were submitted for Illumina sequencing (paired-end 75 bp each side on HiSeq 2500, v4).

### Hi-C data processing, binning, and normalization

The quality check and trimming step of raw data was carried out using BBDuk (version 10/2015)^64^. Setting a minimum read length of 35 bp and a minimum Phred quality score of 20, adapters and low-quality reads were removed while preserving their longest high-quality regions. After the quality check, the reads were processed with TADbit (version 0.2.0.23)^65^, which makes use of the GEM (version 1.7.1) mapper^66^ to iteratively map them against the mouse genome (version mm10). Reads were mapped from 15 bp toward using a step size of 5 bp. The filters used to remove possible artifacts were the following: self-circle, dangling-end, error, extra dangling-end, too short, too large, duplicated, and random breaks. The maximum molecule-length parameter was set at two times the 99.9 percentile of the insert-size distribution, returned by the insert_size from TADbit. The maximum distance of a read to a cleavage site was set to the 99.9 percentile of the insert-size distribution.

An in-house script was used for binning and data normalization. This script imported the HiC_data module of TADbit, read the map files generated after the artifact filtering step, binned the reads into a square matrix of 50 kbp, and stored the matrix into a file in NPZ format (raw matrix). Afterward, HiCExplorer (version 3.3)^67^ was used to normalize with the ICE (Iterative Correction and Eigenvector decomposition). The normalized matrices of standard and Rb were then compared by the log_2_ ratio method using hicCompareMatrix from HiCExplorer to obtain the differential matrices.

Pairwise comparisons between biological replicates derived from the Hi-C experiments were performed using HiCRep (version 1.4), under a smoothing parameter of 5 and a considered distance over 10 Mbp^16,68^. The Y chromosome was excluded from the analysis due to the lower number of interactions detected in our analysis (<1% of the overall detected interactions) and the highly repetitive DNA that characterizes this chromosome. The correlation between 2 replicates was defined as the mean of the 20 correlation scores.

Normalized matrices of standard and Rb mice were transformed into observed/expected matrices using the tool hicTransform from HiCExplorer (version 3.3)^67^. This tool computes the expected matrix as the sum of contacts per genomic distance divided by the maximal possible contacts. Then, the observed matrix was divided by this calculated expected matrix and the observed/expected matrix is obtained.

The observed/expected matrices were compared by the log_2_ ratio method using hicCompareMatrix from HiCExplorer to obtain the differential matrices. Finally, these matrices were plotted by chromosome using a 500-kb resolution.

### Interchromosome/intrachromosome interaction ratio

ICE-normalized data stored in matrices were exported with HiCExplorer to the GInteractions format, which consists of seven columns: chromosome, start and end from bin 1, chromosome, start and end from bin 2, and the amount of interaction. The GInteractions tables were imported in R for further quantification of interchromosome and intrachromosome interactions and plotting.

### Interchromosomal interaction analysis

Using R (version 3.6.1), the GInteraction tables were subset by chromosome, so the analysis of each chromosome interchromosomal interactions could be done individually. Then, the mean of interactions of a given chromosome with others was calculated^16^. Finally, data were plotted as a heatmap where red stands for more interaction in Rb mice and blue in standard mice. The GIinteraction tables subset by chromosome were then plotted as interaction profiles considering the interaction for each genomic position.

The analysis of the intrachromosomal interaction ratio in RS (haploid cells) allowed for a predicted classification of fusions into homozygous or heterozygous by quantifying the interactions of fused chromosomes when compared to chromosomes not involved in Rb fusions. In this manner, we established that fusions 3.8, 6.10, and 5.15 were present in a heterozygous state (ratio between 1.2 and 1.6) while fusions 9.11, 12.13, and 4.14 were present in a homozygous state (ratio around 2).

### Chromosomal-specific interchromosomal interaction analysis

Interchromosomal regions were statistically defined as bins with a standard deviation (Z-score) higher than 2.58. Bin regions were also intersected with BEDTools intersect (version 2.25) against promoter regions (-2 kbp to TSS) from the mouse GENCODE annotation vM14 to obtain the overlapping genes. These genes were then included in a Gene Ontology Enrichment Analysis (GOEA). We used CIRCOS (version 0.69-8)^69^ to plot the genomic positions of the interchromosomal interactions across the mouse genome.

For the analysis of repetitive sequences within interchromosomal regions, repeatMasker^70^ annotation on the *Mus musculus* genome version mm10 was downloaded and parsed to become a BED file. BEDtools (version 2.26) was used to intersect equal-sized bins considered as interchromosomal interaction regions against the RepeatMasker annotation with option -wo, thus counting the number of overlapping bases. All equal-sized bins of the genome were also intersected, and the number of overlapping bases was also counted and considered as the background-repeat profile of the mm10 genome. A Mann–Whitney test was applied to test for significant differences.

### A/B compartments and TAD calling

Analyses were conducted at the genome-wide level. For A/B compartment calling, columns with a low number of counts were filtered out using TADbit, setting the parameter min_count to 10. Since TADbit fits the column count distribution into a polynomial distribution, columns with a number of counts smaller than the first antimode of the distribution, which cannot be smaller than the min_count parameter, are filtered out. Then, the genome-wide matrices were normalized by the expected interactions at a given distance and by visibility by means of one iteration of the ICE method. The correlation analysis was also performed with TADbit. In-house scripts computed A/B compartments from the first eigenvector, using 0 as the threshold to differentiate both compartments and the gene density to label them.

TADs were identified using an in-house script that imported the Chromosome module of TADbit and added the raw and the ICE-normalized matrices of each chromosome separately. Filtered bins, due to low counts, were included to mask them when calling TADs. TAD insulation scores were obtained by first normalizing the different matrices for read depth in order for the scores to be comparable. Each matrix was then scaled to have 100 M reads. Afterward, TAD insulation scores were obtained from the output given by the hicFindTADs program from HiCExplorer.

### Compartment switching

BED files with a resolution of 50 kbp were available from the compartments definition step. Each genomic bin of 50 kbp had its corresponding compartment attributed. Pairwise comparisons between cell types—genome-wide and per-chromosome—were performed; the ratio of compartment switching was calculated as the number of genomic bins with a compartment change (A > B or B > A) divided by the total number of bins. From these files, a matrix file was created with 50-kbp-binned genomic coordinates as rows and cell types as columns, filled by the corresponding compartment labeling in each bin and cell type. Cell-specific A compartments were defined as those bins being compartment A in a cell type and compartment B in the remaining cell types.

### Quantification and statistical analyses

The statistical analyses were performed using R. Statistical parameters and tests are reported in the Figures and Figure Legends. Boxplots are represented in the manner that the middle line is the median, while the lower and upper bounds correspond to the first and third quartiles; the upper whisker extends the largest value within 1.5 times the interquartile range above the 75th percentile and the lower whisker extends to the smallest value within 1.5 times the interquartile range below the 25th percentile.

### Reporting summary

Further information on research design is available in the Nature Research Reporting Summary linked to this article.

## Supplementary information

Supplementary Information

Reporting Summary

## Data Availability

The data that support this study are available from the corresponding author upon reasonable request. Raw and processed Hi-C data from Rb mice generated in the course of this study are available in the NCBI GEO repository under accession number GSE145978. The Hi-C data set from standard mice was retrieved from Vara and collaborators^16^, which is available in the NCBI GEO repository, under the accession number GSE132054. Source data are provided with this paper.

## Source data

Source Data
