# Supplementary material for: The impact of chromosomal fusions on 3D genome folding and recombination in the germ line
Source: Nat Commun. 2021 May 20;12:2981. doi: 10.1038/s41467-021-23270-1 (PMC8137915; doi:10.1038/s41467-021-23270-1)
Supplement: Supplementary file 1 — Supplementary Information [file 41467_2021_23270_MOESM1_ESM.pdf]

Supplementary Information

The Impact of Chromosomal Fusions on 3D Genome Folding and Recombination in the Germ Line

Vara et al.

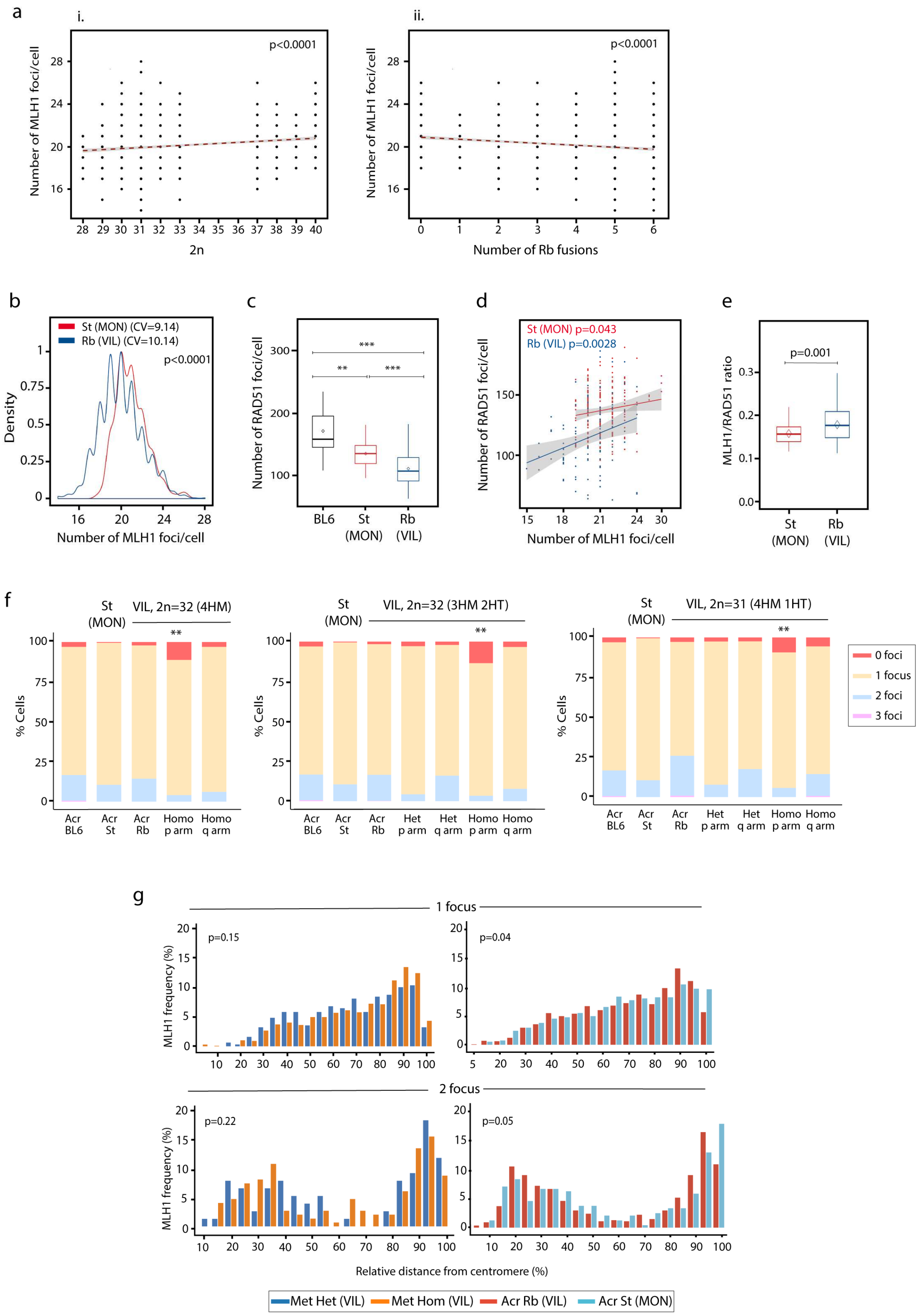

Supplementary Figure 1: **a** Correlation between the number of MLH1 foci per cell (n=1,399 cells) and (i) diploid number (2n) and (ii) the number of Rb fusions in all sampled mice (Spearman, p<0.0001, two-sided). The shaded grey band represents the 95% confidence level interval for predictions from a linear model. Source data are provided as a Source Data file. **b** Density plot showing the distribution of COs in standard (St, red, from

MON population) and Rb mice (Rb, blue, from VIL population) showing greater co-variance in Rb mice (CV=10.14) with respect to their St counterparts (CV=9.14) (Mann-Whitney test, $p<0.0001$, two-sided). Source data are provided as a Source Data file. **c** Number of RAD51 foci detected per cell in laboratory mice (BL6, n=15 cells) and wild-caught St (MON population, n=200 cells) and Rb mice (VIL population, n=122 cells). P-values (Dunn's test, $^{**}p<0.001$; $^{***}p<0.0001$, two-sided) are indicated. Boxplots are presented as mean values +/- SD; center line, median; center diamond, mean. Source data are provided as a Source Data file. **d** Correlation between the number of RAD51 and MLH1 foci per cell in standard (St, red, from MON population, n=200 cells) and Rb mice (Rb, blue, from VIL population, n=122 cells) (Spearman, $p<0.0001$, two-sided). The shaded grey band represents the 95% confidence level interval for predictions from a linear model. Source data are provided as a Source Data file. **e** MLH1/RAD51 ratio in Standard (St, red, from MON population, n=200 cells) and Rb mice (blue, from VIL population, n=122 cells) (Mann-Whitney test, $p=0.001$, two-sided). Boxplots are presented as mean values +/- SD; center line, median; center diamond, mean. Source data are provided as a Source Data file. **f** Proportion of MLH1 foci per arm type showing the proportion of #0, #1, #2 and #3 foci considering different chromosomal arm configurations: Acr BL6, all acrocentric chromosomes of BL6 mice; Acr St, all acrocentric chromosomes of wild standard mice; Acr Rb, acrocentric chromosomes of wild Rb mice; Met Het, Rb chromosomes in a heterozygous state of Rb mice; Met Hom, Rb chromosomes in a homozygous state of Rb mice. Each panel represent different mice from the Viladecans populations with different karyotypes: specimens with four Rb fusions in a homozygous state (HM), specimens with three Rb fusions in a homozygous state plus two fusions in a heterozygous state (HT), and specimens with four Rb fusions in a homozygous state and one in a heterozygous state. The p-arms of homozygous chromosomes show the greatest tendency to lack COs (χ^2 test, $^{**}p<0.0001$, two-sided). Source data are provided as a Source Data file. **g** Distribution of MLH1 foci along individual chromosomal arms with one (left panel) or two (right panel) MLH1 foci. The X-axis represents the relative positions on the chromosomal axes from the centromere (0%) to the distal telomere (100%) (χ^2 test, $p\leq0.05$, two-sided). The Y-axis indicates the frequency of MLH1 foci in each interval of chromosome arm length. Chromosome type legend: Acr St, all acrocentric chromosomes of standard mice from MON population (n=1,014 arms with 1 CO and n=240 arms with 2COs); Acr Rb, acrocentric chromosomes of Rb mice from VIL population (n=1,232 arms with 1 CO and n=542 arms with 2 COs); Met Het, arms of metacentric chromosomes in heterozygous of Rb mice from VIL population (n=308 arms with 1 CO and n=76 arms with 2 COs); Met Hom, arms of metacentric chromosomes in homozygous of Rb mice from VIL population (n=964 arms with 1 CO and n=148 arms with 2 COs). Source data are provided as a Source Data file.

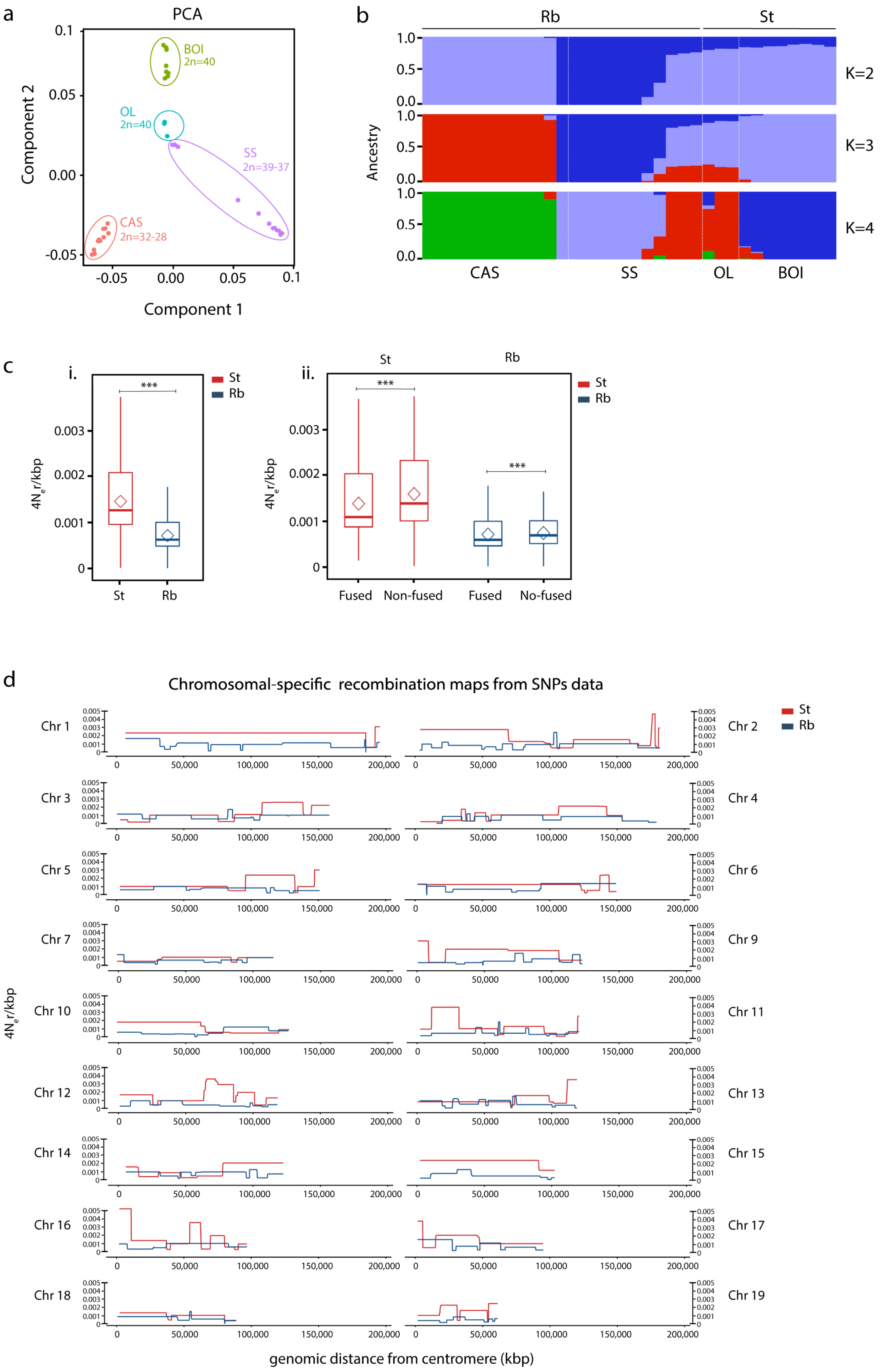

Supplementary Figure 2: **a** Principal component analysis (PCA) of a subset of 34 mice from two standard populations (Castellfollit del Boix – BOI and Olost – OL) and two Rb populations (Sant Sadurní d'Anoia – SS and Castelldefels – CAS) (Supplementary Table

1). Source data are provided as a Source Data file. **b** Plots showing the proportion of inferred ancestry for K = 2 to K = 4 in mice from panel A. Legend: CAS, Castelldefels; SS, Sant Sadurní d'Anoia; OL, Olost; BOI, Castellfollit del Boix). Source data are provided as a Source Data file. **c** Recombination rate estimates expressed as $4N_er$/kbp between standard (n=2,439) and Rb (n=2,437) populations derived from the analysis of SNPs data using LDhelmet. (i) Box plots depict genome-wide recombination rates (expressed as $4N_er$/kbp) for standard (St, red) and Rb mice (blue). Diamonds indicate mean values (Mann-Whitney test ***$p<2.2$ e-16, two-sided). Boxplots are presented as mean values +/- SD; center line, median; center diamond, mean. Source data are provided as a Source Data file. (ii) Differences in recombination estimates (expressed as $4N_er$/kbp) between standard (St, red) and Rb (blue) mice considering chromosomes involved in Rb fusions (3.8; 4.14; 5.15; 6.10; 9.11 and 12.13) and those that are not fused (1, 2, 7, 16, 17, 18, 19 and X). Boxplots are presented as mean values +/- SD; center line, median; center diamond, mean. Source data are provided as a Source Data file. **d** Representation of recombination rates (expressed as $4N_er$/kbp) derived from the analysis of SNPs data using LDhelmet for all autosomal chromosomes. Chromosomal distributions of recombination rates are represented for standard (St) populations in red and for Rb populations in blue. Legend: chr, chromosome. Source data are provided as a Source Data file.

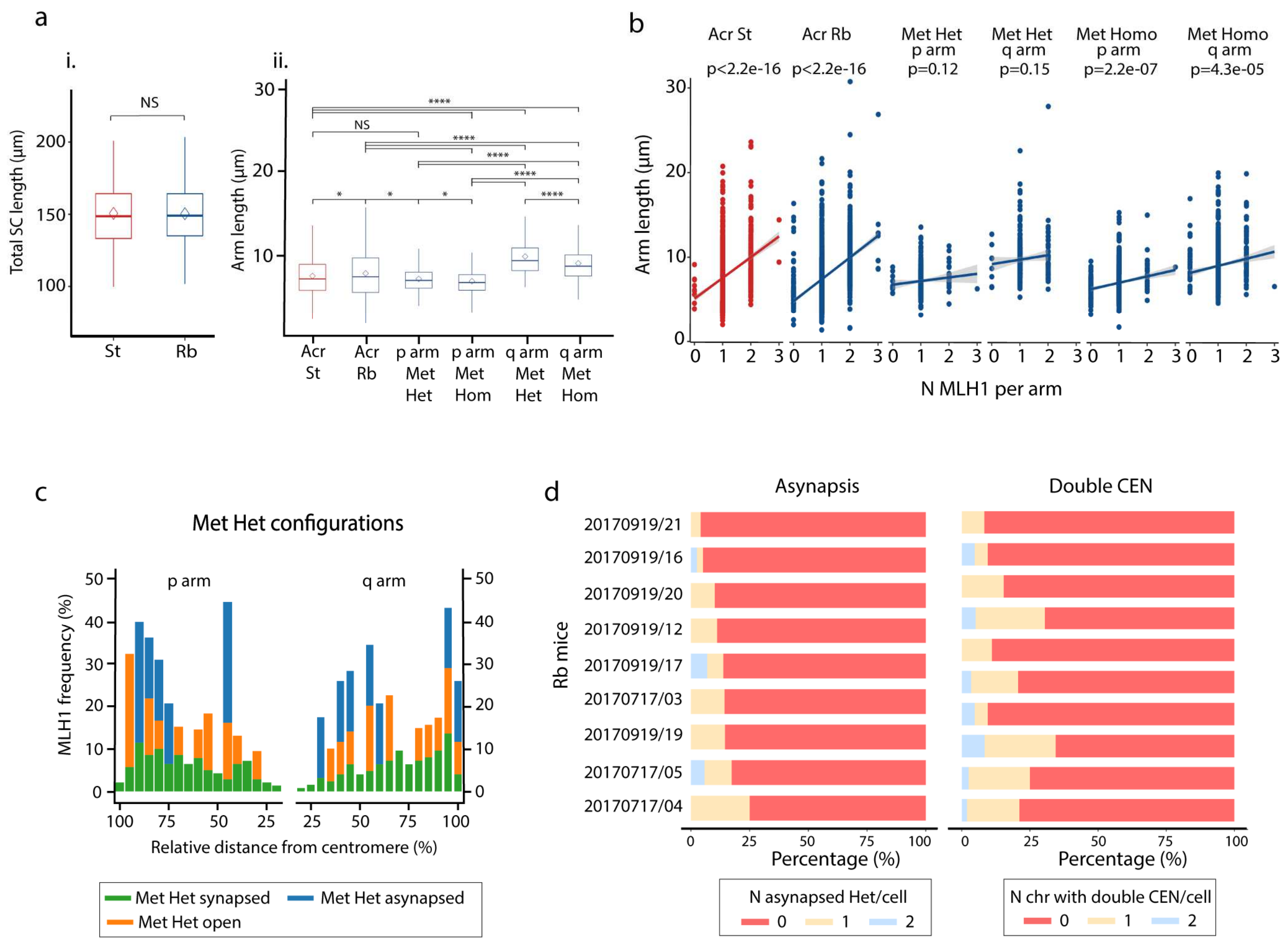

Supplementary Figure 3: **a** Chromosomal axis length analysis. (i) Total axis length (expressed as µm) analysis in standard (St, n=88 cells) and Robertsonian (Rb, n=221 cells) mice from BRbS (see text and Supplementary Table 1 for further details). Mann-Whitney test, p=0.86 (two-sided); NS: non-significant. SC – synaptonemal complex. Boxplots are presented as mean values +/- SD; center line, median; center diamond, mean. Source data are provided as a Source Data file. (ii) Axis length (expressed as µm) analysis detailed by chromosomal arm type: acrocentric standard (Acr St, n=1,140 chromosomal arms), acrocentric Rb (Acr Rb, n=1,711 chromosomal arms), p chromosomal arms (n=198) and q chromosomal arms (n=198) of metacentrics in a heterozygous state (Met Het) and p chromosomal arms (n=643) and q chromosomal arms (n=643) in homozygous state (Met Hom). Wilcoxon's rank sum test followed by Dunn's tests adjusted by Bonferroni comparisons are shown: NS-non significant, *p-value<0.05, ****p-value<0.0001 (two-sided). Boxplots are presented as mean values +/- SD; center line, median; center diamond, mean. Source data are provided as a Source Data file. **b** Correlation between axis length (expressed as µm) and the number of MLH1 foci (#0, #1, #2 and #3 foci) per chromosomal arm for each chromosome type: acrocentric standard (Acr St, n=1,710 chromosomal arms), acrocentric Rb (Acr Rb, n= 2,423 chromosomal arms), p and q arms of metacentrics in a heterozygous state (Met Het, n=262 p chromosomal arms and n=262 q chromosomal arms)

and in a homozygous state (Met Hom, n=835 p chromosomal arms and n=835 q chromosomal arms) (Spearman test, p<0.0001, two-sided). The shaded grey band represents the 95% confidence level interval for predictions from a linear model. Source data are provided as a Source Data file. **c** Distribution of MLH1 foci along individual chromosomal arms according to synapsis state (synapsed, open and asynapsed) of heterozygous metacentrics. The X-axis represents the positions on the chromosomal axes from the centromeric end (black dot) to the distal telomere. The Y-axis indicates the frequency of MLH1 foci for each 10% interval of chromosomal length. Source data are provided as a Source Data file. **d** Proportion of asynapsed heterozygous Rb fusions per cell (left panel) and proportion of homozygous Rb fusions with double centromeric signals (CEN) in each Rb mouse analyzed (Supplementary Table 1, n= 573 cells). Source data are provided as a Source Data file.

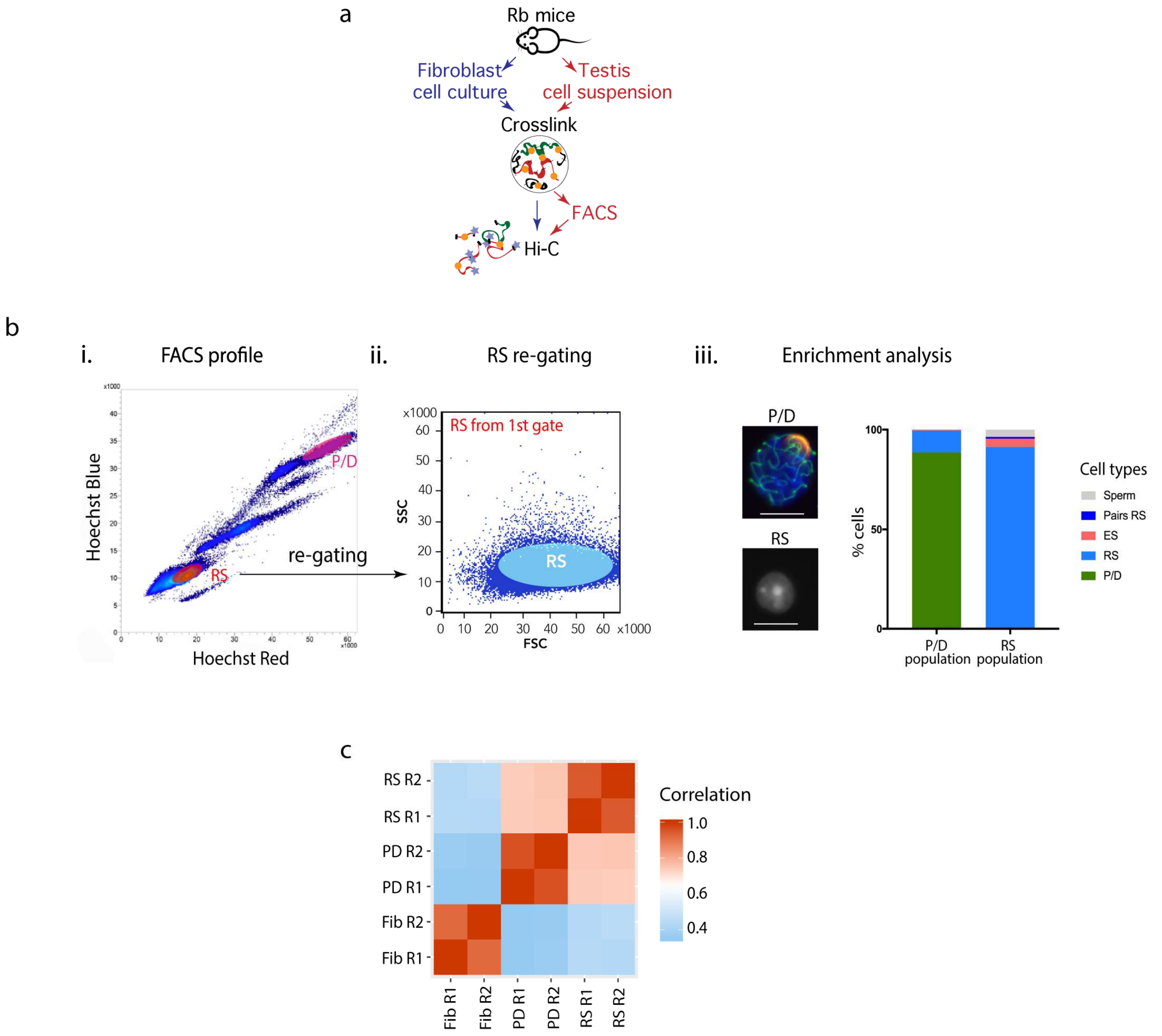

Supplementary Figure 4: **a** Representation of the workflow for the Hi-C experiments. **b** Representative FACS profiles representing Hoechst Blue (UV355-460/50) and Hoechst Red (UV355-670/30). (i) FACS profile of a Rb mouse testis where gates for pachytene/diplotene (P/D) and round spermatids (RS) were placed and sorted accordingly. (ii) Re-gating strategy for round spermatids, where cells gated as shown in (i) were re-analyzed and sorted by their FSC vs. SSC profile, yielding higher purity. (iii) FACS enrichment analysis. Left panel: Insets show immunofluorescence of examples of the cell type sorted in the P/D (DAPI in blue, SYCP3 in green and γH2AX in red) and round spermatids (DAPI in grey) populations (scale bar = 10 µm). In the case of P/D, DAPI is represented in blue, SYCP3 in green and gamma-H2AX in red. In round spermatids DAPI is represented in grey. Immunofluorescence replicates, n=3. Enrichment values obtained for P/D and RS populations after FACS, both circa 90%. Legend: RS, round spermatids; ES: elongated

spermatids; P/D, pachynema/diplonema. Source data are provided as a Source Data file. **c** Heatmap showing the correlation values among replicates based on the pairwise similarity score calculated using HiCRep (see Methods). Legend: PD, Pachytene/Diplotene; RS, Round Spermatids; R#, replicates. Source data are provided as a Source Data file.

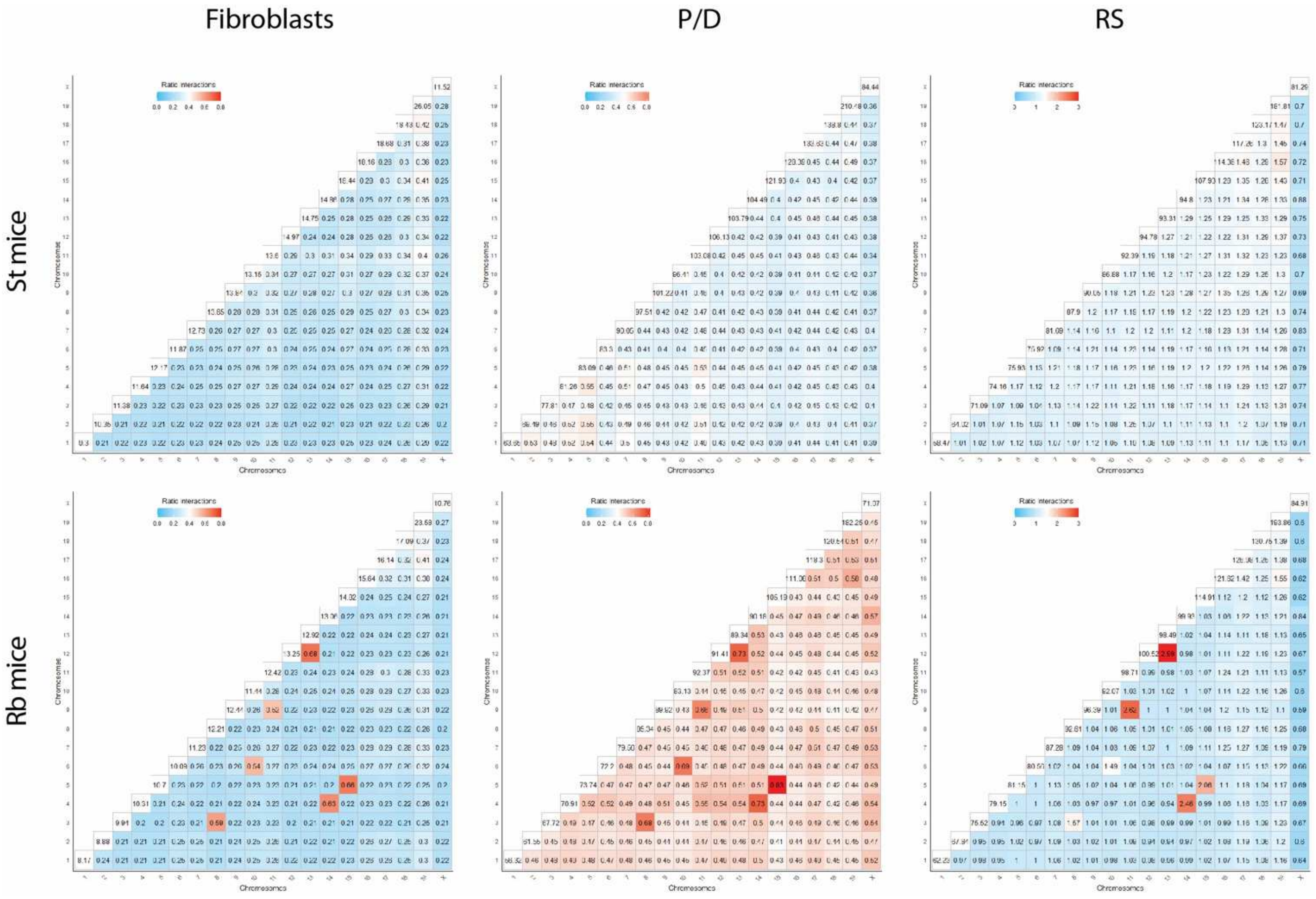

Supplementary Figure 5: Heatmaps depicting mean values of inter-chromosomal interaction ratios per chromosome in each cell type in standard (St) and Rb mice. Legend – P/D: Pachytene/Diplotene, RS: Round Spermatids. Source data are provided as a Source Data file.

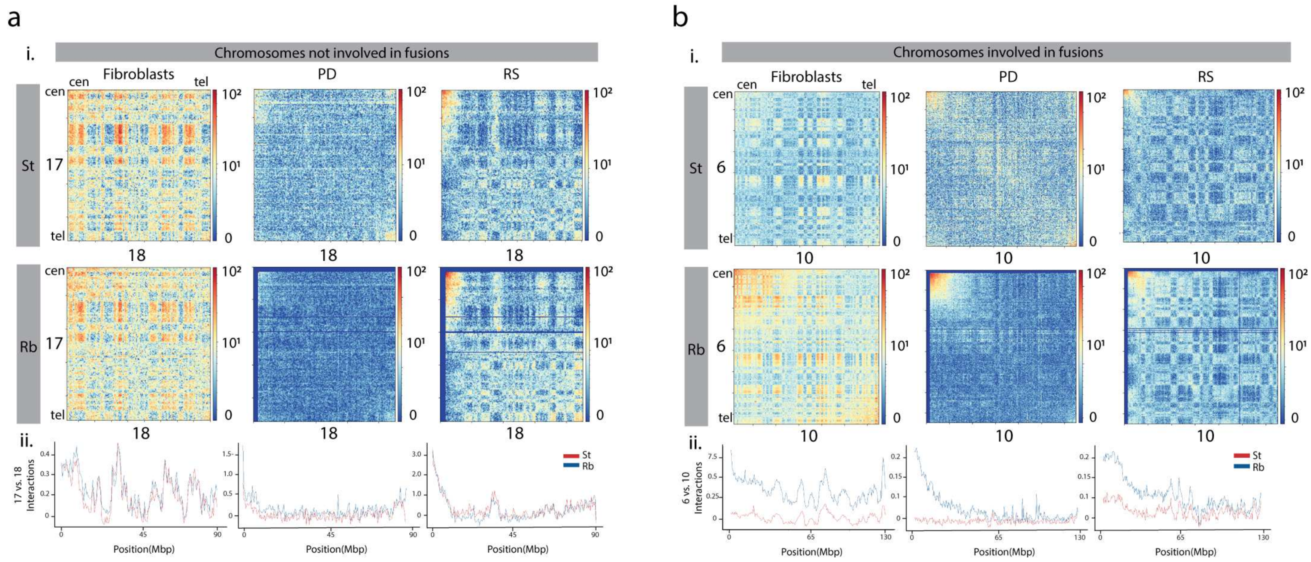

Supplementary Figure 6: Examples of interaction patterns between chromosomes. **a** Chromosomes not involved in fusions. **b** Chromosomes involved in fusions. (i) Interaction heatmaps representing chromosomes 17 and 18 (not involved in fusions) and chromosomes 6 and 10 (involved in fusions). Maps are represented for both standard (St) and Rb mice. In chromosomes 6 and 10, the fusion becomes evident in interaction maps from Rb mice, with high interaction in the pericentromeric region of the chromosomes (0 to 3 Mbps from the centromere). Source data are provided as a Source Data file. (ii) Interaction plots for fibroblasts, P/D and RS for chromosomes not involved (17 and 18) and involved in fusions (6 and 10). Source data are provided as a Source Data file.

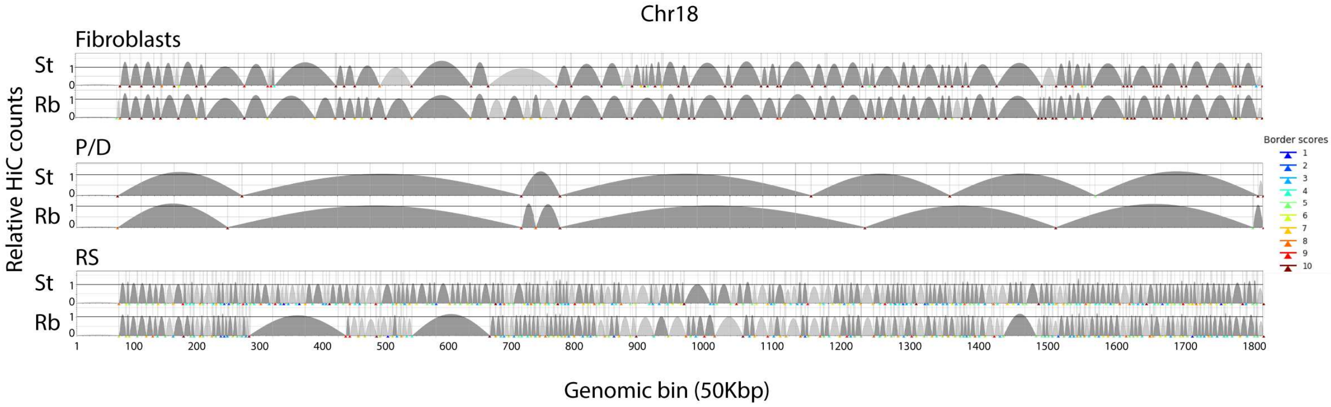

Supplementary Figure 7: TAD border alignments along chromosome 18 between standard (St) and Rb mice in all cell types included in the study (fibroblasts, pachynema/diplonema – P/D and round spermatids – RS). Dark grey arches represent TADs with higher (light grey lower) than expected intra-TAD interactions. TAD border robustness (from 1 to 10) is represented by a color gradient. Source data are provided as a Source Data file.

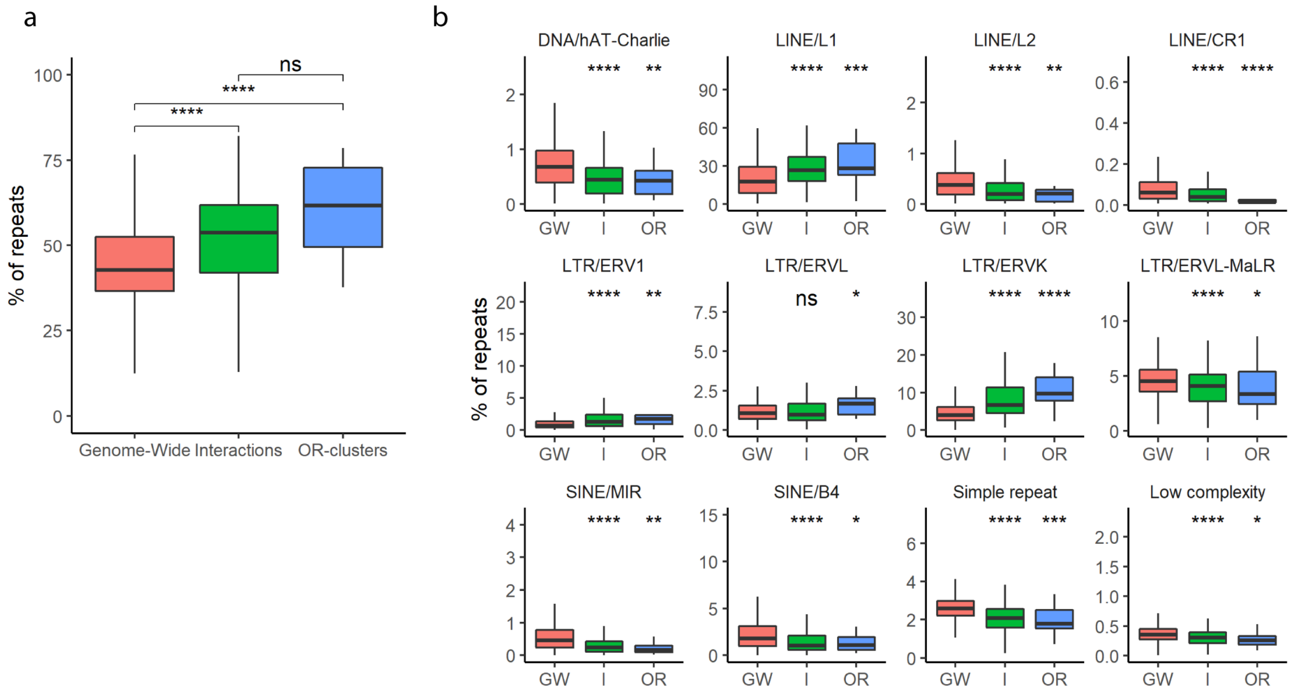

Supplementary Figure 8: **a** Representation of repetitive sequences represented in the mouse genome (‘genome-wide’, n=5,334) when compared to newly detected inter-chromosomal interactions (n=236) in primary spermatocytes (pachynema/diplonema, P/D) in Rb mice and olfactory receptor (OR) clusters (n=223) (Wilcoxon test, ****p≤ 0.0001, two-sided). Source data are provided as a Source Data file. **b** Detailed analysis of the different types of repeats present in sensory perception bins when compared to the rest of the mouse genome (Wilcoxon test, ****p≤.0001, ***p≤0.001, **p≤0.01, *p≤0.05, two-sided). Legend – GW: genome wide (n=5,286 for DNA/hAT-Charlie, n= 4,554 for LINE/CR1, n=5,334 for LINE/L1, n=5,157 for LINE/L2, n=5,282 for LTR/ERV1, n=5,334 for LTR/ERVK, n=5,325 for LTR/ERVL, n=5,332 for LTR/ERVL-MaLR, n=5,333 for Low complexity, n=5,325 for SINE/B4, n=5,021 for SINE/MIR, n=5,334 for Simple repeat) I: inter-chromosomal interactions (n=222 for DNA/hAT-Charlie, n=160 for LINE/CR1, n=236 for LINE/L1, 211 for LINE/L2, n=233 for LTR/ERV1, n=236 for LTR/ERVK, n=232 for LTR/ERVL, n=236 for LTR/ERVL-MaLR, n=236 for Low complexity, n=234 for SINE/B4, n=194 for SINE/MIR, n=236 for Simple repeat), OR: olfactory receptor clusters (n=17 for DNA/hAT-Charlie, n=13 for LINE/CR1, n=17 for LINE/L1, n=15 for LINE/L2, n=17 for LTR/ERV1, n=17 for LTR/ERVK, n=17 for LTR/ERVL, n=17 for LTR/ERVL-MaLR, n=17 for Low complexity, n=17 for SINE/B4, n=14 for SINE/MIR, n=17 for Simple repeat), ns: non-significant. Boxplots are presented as mean values +/- SD. Source data are provided as a Source Data file.

Supplementary Table 1: List of all wild-caught mice included in the study. Populations sampled, sex, individual ID, diploid number (2n) and Rb fusions for each mouse from the Barcelona Rb system. The analysis performed on each individual is detailed. Abbreviations: HM – fusions in a homozygous state; HT– fusions in a heterozygous state; CO –crossovers; DSBs – double strand breaks; SC – synaptonemal complex; R# – replicates; N.A. – Not analyzed.

					CO analysis		DSBs analysis						
POPULATION	SEX	ID	2n	FUSIONS	N	Mean MLH1/cell	N	Mean RAD51/cell	SC length and CO/arm analysis	Double CEN signal analysis	Sperm analysis	SNPs	HiC
Caldes de Montbuí	MALE	01_ST_20160316_01	40		25	20.84	25	123.32	yes	n.a.	n.a.		
Caldes de Montbuí	MALE	01_ST_20160316_02	40		25	20.52	25	140.68	yes	n.a.	n.a.		
Caldes de Montbuí	MALE	01_ST_20160316_03	40		25	20.72	25	130.64	yes	n.a.	n.a.		
Caldes de Montbuí	MALE	01_ST_20160316_04	40		25	21.32	25	128.40	yes	n.a.	n.a.		
Caldes de Montbuí	MALE	01_ST_20160316_05	40		25	20.84	25	127.60	yes	n.a.	n.a.		
Caldes de Montbuí	MALE	01_ST_20160316_07	40		25	22.12	25	143.40	n.a.	n.a.	n.a.		
Caldes de Montbuí	MALE	01_ST_20160316_08	40		25	21.20	25	150.28	yes	n.a.	n.a.		
Castellfollit del Boix	MALE	02_ST_1000	40		25	20.68	n.a.	n.a.	n.a.	n.a.	yes		
Castellfollit del Boix	FEMALE	02_ST_1001	40		n.a.	n.a.	n.a.	n.a.	n.a.	n.a.	n.a.	yes	
Castellfollit del Boix	MALE	02_ST_1019	40		n.a.	n.a.	n.a.	n.a.	n.a.	n.a.	n.a.	yes	
Castellfollit del Boix	MALE	02_ST_1020	40		20	20.90	n.a.	n.a.	n.a.	n.a.	yes	yes	
Castellfollit del Boix	MALE	02_ST_1021	40		21	20.81	n.a.	n.a.	n.a.	n.a.	yes		
Castellfollit del Boix	MALE	02_ST_1024	40		25	20.16	n.a.	n.a.	n.a.	n.a.	n.a.	yes	
Castellfollit del Boix	MALE	02_ST_979	40		26	20.27	n.a.	n.a.	n.a.	n.a.	n.a.	yes	
Castellfollit del Boix	MALE	02_ST_980	40		22	20.68	n.a.	n.a.	n.a.	n.a.	yes		
Castellfollit del Boix	FEMALE	02_ST_984	40		n.a.	n.a.	n.a.	n.a.	n.a.	n.a.	n.a.	yes	
Castellfollit del Boix	MALE	02_ST_998	40		21	20.57	n.a.	n.a.	n.a.	n.a.	yes	yes	
Castellfollit del Boix	MALE	02_ST_999	40		14	20.57	n.a.	n.a.	n.a.	n.a.	yes	yes	
Olost	MALE	03_ST_10L	40		n.a.	n.a.	n.a.	n.a.	n.a.	n.a.	n.a.	yes	
Olost	FEMALE	03_ST_11L	40		n.a.	n.a.	n.a.	n.a.	n.a.	n.a.	n.a.	yes	
Olost	MALE	03_ST_14L	40		n.a.	n.a.	n.a.	n.a.	n.a.	n.a.	n.a.	yes	
Sant Sadurni d'Anoia	MALE	04_Rb_SS12	36	1 HM, 2 HT (4.14, 9.11, 12.13)	n.a.	n.a.	n.a.	n.a.	n.a.	n.a.	n.a.	yes	
Sant Sadurni d'Anoia	MALE	04_Rb_SS13	38	1 HM (12.13)	n.a.	n.a.	n.a.	n.a.	n.a.	n.a.	n.a.	yes	
Sant Sadurni d'Anoia	MALE	04_Rb_SS18	37	3 HT (4.14, 9.11, 12.13)	47	20.15	n.a.	n.a.	n.a.	n.a.	n.a.	yes	
Sant Sadurni d'Anoia	MALE	04_Rb_SS19	37	1 HM, 1 HT (4.14, 9.11)	n.a.	n.a.	n.a.	n.a.	n.a.	n.a.	n.a.	yes	
Sant Sadurni d'Anoia	MALE	04_Rb_SS20	39	1 HT (12.13)	27	20.26	n.a.	n.a.	n.a.	n.a.	n.a.	yes	
Sant Sadurni d'Anoia	MALE	04_Rb_SS22	39	1 HT (4.14)	n.a.	n.a.	n.a.	n.a.	n.a.	n.a.	n.a.	yes	
Sant Sadurni d'Anoia	MALE	04_Rb_SS2	35	1 HT, 2 HM (4.14, 9.11, 12.13)	n.a.	n.a.	n.a.	n.a.	n.a.	n.a.	n.a.	yes	
Sant Sadurni d'Anoia	FEMALE	04_Rb_SS4	38	2 HT (4.14, 12.13)	n.a.	n.a.	n.a.	n.a.	n.a.	n.a.	n.a.	yes	
Sant Sadurni d'Anoia	MALE	04_Rb_SS5	37	1 HM, 1 HT (4.14, 12.13)	n.a.	n.a.	n.a.	n.a.	n.a.	n.a.	n.a.	yes	
Sant Sadurni d'Anoia	MALE	04_Rb_SS6	37	1 HT, 1 HM (4.14, 9.11)	40	19.80	n.a.	n.a.	n.a.	n.a.	n.a.	yes	
Sant Sadurni d'Anoia	MALE	04_Rb_SS7	38	2 HT (4.14, 9.11)	46	19.72	n.a.	n.a.	n.a.	n.a.	n.a.	yes	
Sant Sadurni d'Anoia	MALE	04_Rb_SS8	37	3 HT (4.14, 9.11, 12.13)	46	20.57	n.a.	n.a.	n.a.	n.a.	n.a.		
Viladecans	MALE	05_Rb_20170717_02	33	3 HM, 1HT (3.8, 4.14, 5.15, 6.10, 9.11, 12.13)	25	20.68	20	142.45	n.a.	n.a.	n.a.		R #1
Viladecans	MALE	05_Rb_20170717_03	32	3 HM 2HT (3.8, 4.14, 5.15, 6.10, 9.11, 12.13)	32	20.38	20	134.7	yes	yes	n.a.		R #1
Viladecans	MALE	05_Rb_20170717_04	31	4 HM, 1HT (3.8, 4.14, 5.15, 6.10, 9.11, 12.13)	25	20.00	n.a.	n.a.	n.a.	yes	n.a.		R #1
Viladecans	MALE	05_Rb_20170717_05	32	3 HM, 2HT (3.8, 4.14, 5.15, 6.10, 9.11, 12.13)	33	20.52	n.a.	n.a.	n.a.	yes	n.a.		R #1
Viladecans	MALE	05_Rb_20170721_01	32	4 HM (3.8, 4.14, 5.15, 6.10, 9.11, 12.13)	25	20.89	n.a.	n.a.	yes	n.a.	n.a.		
Viladecans	MALE	05_Rb_20170727_01	33	3 HM, 1HT (3.8, 4.14, 5.15, 6.10, 9.11, 12.13)	41	20.12	n.a.	n.a.	n.a.	n.a.	n.a.		
Viladecans	MALE	05_Rb_20170906_03	32	4 HM (3.8, 4.14, 5.15, 6.10, 9.11, 12.13)	25	19.16	n.a.	n.a.	yes	n.a.	n.a.		
Viladecans	MALE	05_Rb_20170919_12	31	4 HM, 1HT (3.8, 4.14, 5.15, 6.10, 9.11, 12.13)	15	19.47	21	90.29	n.a.	yes	n.a.		R #2
Viladecans	MALE	05_Rb_20170919_16	31	4 HM, 1HT (3.8, 4.14, 5.15, 6.10, 9.11, 12.13)	32	19.09	21	91.90	n.a.	yes	n.a.		R #2
Viladecans	MALE	05_Rb_20170919_17	32	3 HM, 2HT (3.8, 4.14, 5.15, 6.10, 9.11, 12.13)	34	20.12	n.a.	n.a.	yes	yes	n.a.		
Viladecans	MALE	05_Rb_20170919_19	31	4 HM, 1HT (3.8, 4.14, 5.15, 6.10, 9.11, 12.13)	74	21.25	n.a.	n.a.	yes	yes	n.a.		R #2
Viladecans	MALE	05_Rb_20170919_20	31	4 HM, 1HT (3.8, 4.14, 5.15, 6.10, 9.11, 12.13)	48	19.97	24	97.33	yes	yes	n.a.		R #2
Viladecans	MALE	05_Rb_20170919_21	32	3HM, 2HT (3.8, 4.14, 5.15, 6.10, 9.11, 12.13)	25	18.96	16	113.94	yes	n.a.	n.a.		
Viladecans	MALE	05_Rb_946	31	3 HM, 3 HT (3.8, 4.14, 5.15, 6.10, 9.11, 12.13)	22	18.86	n.a.	n.a.	n.a.	n.a.	yes		
Viladecans	MALE	05_Rb_947	32	3 HM, 2HT (3.8, 4.14, 6.10, 9.11, 12.13)	55	19.45	n.a.	n.a.	n.a.	n.a.	n.a.		
Castelldefells	MALE	06_Rb_922	29	4 HM, 1 HT (4.14, 5.15, 6.10, 9.11, 12.13)	22	18.95	n.a.	n.a.	n.a.	n.a.	yes		
Castelldefells	MALE	06_Rb_923	28	6 HM (3.8, 4.14, 5.15, 6.10, 9.11, 12.13)	18	19.39	n.a.	n.a.	n.a.	n.a.	n.a.	yes	
Castelldefells	MALE	06_Rb_954	30	4 HM, 2 HT (3.8, 4.14, 5.15, 6.10, 9.11, 12.13)	39	21.82	n.a.	n.a.	n.a.	n.a.	yes	yes	R #1 & #2
Castelldefells	MALE	06_Rb_955	32	3 HM, 2 HT (4.14, 5.15, 6.10, 9.11, 12.13)	40	19.93	n.a.	n.a.	n.a.	n.a.	yes	yes	
Castelldefells	MALE	06_Rb_956	30	4 HM, 2 HT (3.8, 4·14, 5.15, 6.10, 9.11, 12.13)	37	19.62	n.a.	n.a.	n.a.	n.a.	yes		
Castelldefells	FEMALE	06_Rb_960	28	5 HM (4.14, 5.15, 6.10, 9.11, 12.13)	n.a.	n.a.	n.a.	n.a.	n.a.	n.a.	n.a.	yes	
Castelldefells	FEMALE	06_Rb_963	29	4 HM, 3 HT (3.8, 4.14, 5.15, 6.10, 9.11, 12.13, 7.17)	n.a.	n.a.	n.a.	n.a.	n.a.	n.a.	n.a.	yes	
Castelldefells	MALE	06_Rb_967	29	5 HM, 1 HT (3.8, 4.14, 5.15, 6.10, 9.11, 12.13)	44	19.95	n.a.	n.a.	n.a.	n.a.	yes	yes	
Castelldefells	MALE	06_Rb_968	30	4 HM, 2 HT (3.8, 4·14, 5.15, 6.10, 9.11, 12.13)	27	19.85	n.a.	n.a.	n.a.	n.a.	yes	yes	
Castelldefells	MALE	06_Rb_970	30	4 HM, 2 HT (3.8, 4.14, 5.15, 6.10, 9.11, 12.13)	19	19.00	n.a.	n.a.	n.a.	n.a.	n.a.	yes	
Castelldefells	MALE	06_Rb_971	30	4 HM, 2 HT (3.8, 4.14, 5.15, 6.10, 9.11, 12.13)	34	18.74	n.a.	n.a.	n.a.	n.a.	yes		
Castelldefells	MALE	06_Rb_972	31	3 HM, 3 HT (3.8, 4.14, 5.15, 6.10, 9.11, 12.13)	16	18.13	n.a.	n.a.	n.a.	n.a.	n.a.	yes	
Castelldefells	MALE	06_Rb_CS10	31	4 HM, 1 HT (3.8, 4.14, 6.10, 9.11, 12.13)	n.a.	n.a.	n.a.	n.a.	n.a.	n.a.	n.a.	yes	
Castelldefells	FEMALE	06_Rb_CS13	30	4 HM, 2 HT (3.8, 4.14, 5.15, 6.10, 9.11, 12.13)	n.a.	n.a.	n.a.	n.a.	n.a.	n.a.	n.a.	yes	
Castelldefells	FEMALE	06_Rb_CS14	30	4 HM, 2 HT (3.8, 4.14, 5.15, 6.10, 9.11, 12.13)	n.a.	n.a.	n.a.	n.a.	n.a.	n.a.	n.a.	yes	

Supplementary Table 2: Genetic diversity. Representation of number of individuals (n), number of alleles (Na), allelic richness (A_r), observed heterozygosity (H_O), expected heterozygosity (H_e), inbreeding coefficient (F_{IS}), and nucleotide diversity (pi).

Group	n	Na	A_r	H_o	H_e	F_{IS}	pi
Standard	11	95006	95006	0.1576	0.1680	0.1048	0.1760
Sant Sadurní	11	91738	91738	0.1563	0.1483	-0.0055	0.1554
Castelldefels	12	93161	92862	0.1561	0.1536	0.0268	0.1602

Supplementary Table 3: F_{ST} values between different populations. N.A.: Not analyzed.

	CASTELLFOLLIT	OLOST	CASTELLDEFELS
**OLOST**	0.1384	N.A.	N.A.
**CASTELLDEFELS**	0.1764	0.1656	N.A.
**SANT SADURNI**	0.1787	0.1736	0.1673

Supplementary Table 4: **F_{ST} values between different populations according to the presence of Rb fusions.** In bold, F_{ST} values when including chromosomes involved in Rb fusions (3.8; 4.14; 5.15; 6.10; 9.11 and 12.13). Unbolded F_{ST} values correspond to comparisons including chromosomes not involved in Rb fusions (1, 2, 7, 10, 16, 17, 18, 19 and X). N.A.: Not analyzed.

	CASTELLFOLLIT	OLOST	CASTELLDEFELS	SANT SADURNI
CASTELLFOLLIT	N.A.	**0.1382**	**0.1781**	**0.1783**
OLOST	0.1362	N.A.	**0.1661**	**0.1709**
CASTELLDEFELS	0.1708	0.1621	N.A.	**0.1689**
SANT SADURNI	0.1780	0.1785	0.1630	N.A.

Supplementary Table 5: Hi-C quality metrics per cell type in Rb mice. P/D: Pachynema/diplonema; RS: Round spermatids.

Information per cell type	Fibroblasts Rb	P/D Rb	RS Rb
Raw (base pairs)	499,788,826	451,041,073	450,256,473
Trimmed q20 (pairs)	473,512,735	424,878,135	423,115,492
Mapped uniquely (pairs)	338,880,234	296,839,992	297,248,047
Self-circle (% relative uniquely mapped)	0.16	0.24	0.32
Dangling-end (% relative uniquely mapped)	3.29	3.22	0.23
Error (% relative uniquely mapped)	2.87	2.72	0.11
Extra dangling-end (% relative uniquely mapped)	1.86	3.75	6.89
Too short (% relative uniquely mapped)	4.43	7.81	10.81
Too large (% relative uniquely mapped)	0.01	0.00	0.00
Duplicated (% relative uniquely mapped)	15.30	16.51	10.90
Random breaks (% relative uniquely mapped)	0.42	0.51	0.04
Total valid (base pairs)	255,930,322	253,785,807	255,264,346
Total valid (% relative to Raw)	51.21	56.27	56.69
Total valid (% relative to Trimmed)	54.05	59.73	60.33
Total valid (% relative to Mapped uniquely)	75.52	85.50	85.88

Supplementary Table 6: Hi-C quality metrics per individual libraries.

Information per library	HH3YTBBXX_6_7 (95401)	HH52MBBXX_2_7 (95401)	HHVVNBBXX_3_14 (95402)	HHW7MBBXX_3_14 (95402)	CBLPLANXX_4_12_1 (P7Rb)	CBLPLANXX_5_14_1 (P11Rb)
Raw (pairs)	115128742	143421403	177632905	63605776	209791352	225385285
Trimmed q20 (pairs)	110564127	136600866	166180747	60166995	203153522	217914691
Ratio Trimmed/Raw	0.96	0.95	0.94	0.95	0.97	0.97
Mapped uniquely (pairs)	81919920	101227127	114239111	41494076	141603450	150746989
Mapping efficiency (%)	74.09	74.10	68.74	68.96	69.70	69.18
Self-circle	80914	101630	245031	88014	273789	319209
Dangling-end	1460724	1832200	5620100	2057441	234781	204949
Error	956811	1759395	4880873	1735209	84316	114882
Extra dangling-end	860499	1078099	3060511	1085005	5581191	7090260
Too short	2047426	2511069	7419022	2705306	9953681	10435096
Too large	8801	10886	5275	1880	3865	4404
Duplicated	7507404	9414380	23861232	6585527	12592497	9848530
Random breaks	167942	208250	789805	366376	39701	38357
Total valid	69409690	85069608	73182181	28268843	114744489	124472314
Total valid (% relative to Raw)	60.29	59.31	41.2	44.44	54.69	55.23
Total valid (% relative to Trimmed)	62.78	62.28	44.04	46.98	56.48	57.12
Total valid (% relative to Mapped uniquely)	84.73	84.04	64.06	68.13	81.03	82.57

CBMVPANXX_4_12_1 (P7Rb)	CCYYKANXX_3_14_1 (P7Rb)	CCYYKANXX_3_15_1 (P11Rb)	CD1RWANXX_3_15_1 (P11Rb)	CD1RWANXX_4_14_1 (P7Rb)
30401344	67073167	71546194	153324994	143775210
28895159	61246874	65036146	140164655	131582580
0.95	0.91	0.91	0.91	0.92
20070529	42988025	46502151	99998907	92177988
69.46	70.19	71.50	71.34	70.05
38453	62978	68807	148676	133637
33509	51368	30927	65999	110901
11440	23840	17109	36111	50165
788408	1387958	1973359	4424762	2631305
1415346	2283560	2410001	5205212	4920692
531	1376	1545	3305	2880
269464	462407	351747	1553969	2075878
5671	10288	7292	14256	29066
17611261	38841899	41803930	88988102	82588158
57.93	57.91	58.43	58.04	57.44
60.95	63.42	64.28	63.49	62.77
87.75	90.36	89.9	88.99	89.6
