# Supplementary material for: The impact of chromosomal fusions on 3D genome folding and recombination in the germ line
Source: Nat Commun. 2021 May 20;12:2981. doi: 10.1038/s41467-021-23270-1 (PMC8137915; doi:10.1038/s41467-021-23270-1)
Supplement: Supplementary file 2 — Reporting Summary [file 41467_2021_23270_MOESM2_ESM.pdf]

nature research

Corresponding author(s): Aurora Ruiz-Herrera

Last updated by author(s): Mar 22, 2021

Reporting Summary

Nature Research wishes to improve the reproducibility of the work that we publish. This form provides structure for consistency and transparency in reporting. For further information on Nature Research policies, see our Editorial Policies and the Editorial Policy Checklist.

Statistics

For all statistical analyses, confirm that the following items are present in the figure legend, table legend, main text, or Methods section.

n/a	Confirmed	
☐	☒	The exact sample size (*n*) for each experimental group/condition, given as a discrete number and unit of measurement
☒	☐	A statement on whether measurements were taken from distinct samples or whether the same sample was measured repeatedly
☐	☒	The statistical test(s) used AND whether they are one- or two-sided<br>*Only common tests should be described solely by name; describe more complex techniques in the Methods section.*
☒	☐	A description of all covariates tested
☒	☐	A description of any assumptions or corrections, such as tests of normality and adjustment for multiple comparisons
☐	☒	A full description of the statistical parameters including central tendency (e.g. means) or other basic estimates (e.g. regression coefficient) AND variation (e.g. standard deviation) or associated estimates of uncertainty (e.g. confidence intervals)
☐	☒	For null hypothesis testing, the test statistic (e.g. *F*, *t*, *r*) with confidence intervals, effect sizes, degrees of freedom and *P* value noted<br>*Give P values as exact values whenever suitable.*
☒	☐	For Bayesian analysis, information on the choice of priors and Markov chain Monte Carlo settings
☒	☐	For hierarchical and complex designs, identification of the appropriate level for tests and full reporting of outcomes
☒	☐	Estimates of effect sizes (e.g. Cohen's *d*, Pearson's *r*), indicating how they were calculated

Our web collection on statistics for biologists contains articles on many of the points above.

Software and code

Policy information about availability of computer code

Data collection

non software was used

Data analysis

ADMIXTURE (version 1.3) (https://dalexander.github.io/admixture/index.html)
BBDuk (version 10/2015) (https://sourceforge.net/projects/bbmap/)
BD FACSTM software (version 1.0)
BEDtools (version 2.26) (https://github.com/arq5x/bedtools2)
CIRCOS (version 0.69-8) (http://circos.ca/)
GEM (version 1.7.1) (https://sourceforge.net/projects/gemlibrary/)
HiCExplorer (version 3.3) (https://github.com/deeptools/HiCExplorer)
HiCRep (version 1.3) (https://github.com/TaoYang-dev/hicrep)
Hierfstat (version v0.04-22) (https://www.rdocumentation.org/packages/hierfstat)
JMP package version 5.1.2, SAS Institute Inc
LDhelmet (version 1.10) (https://sourceforge.net/projects/ldhelmet/)
LDpop (version 1.0.0) (https://github.com/CBIIT/nci-webtools-dceg-linkage/)
Micromeasure 3.3 software
PLINK (version 1.9) (https://www.cog-genomics.org/plink/)
Pophelper (version 2.3.0) (https://github.com/royfrancis/pophelper/releases)
R package (version 3.6.1) (https://www.r-project.org/)
R package Pophelper v2.3.0
Repeat Masker (version 4.0.0) (https://www.repeatmasker.org/)
SHAPEIT (version 2.904.3.10.0) (https://mathgen.stats.ox.ac.uk/genetics_software/shapeit/shapeit.html)
StAMPP (version 1.6.1) (https://cran.r-project.org/web/packages/StAMPP/index.html)
TADbit (version 0.2.0.23) (https://github.com/3DGenomes/TADbit)

VCFtools (version 162v0.1.16) (https://vcftools.github.io/documentation.html)

For manuscripts utilizing custom algorithms or software that are central to the research but not yet described in published literature, software must be made available to editors and reviewers. We strongly encourage code deposition in a community repository (e.g. GitHub). See the Nature Research guidelines for submitting code & software for further information.

Data

Policy information about availability of data

All manuscripts must include a data availability statement. This statement should provide the following information, where applicable:

- Accession codes, unique identifiers, or web links for publicly available datasets
- A list of figures that have associated raw data
- A description of any restrictions on data availability

We have included in the text a data availability statement providing the following information:

1. Accession codes, unique identifiers, or web links for publicly available datasets: The Hi-C dataset from standard mice is available in the NCBI GEO repository, accession number GSE132054. Raw and processed Hi-C data from Rb mice can be found under accession number GSE145978.
2. A mention to source data provided. Each raw data associated with figures is mentioned in the figure legends.

Field-specific reporting

Please select the one below that is the best fit for your research. If you are not sure, read the appropriate sections before making your selection.

☒ Life sciences ☐ Behavioural & social sciences ☐ Ecological, evolutionary & environmental sciences

For a reference copy of the document with all sections, see nature.com/documents/nr-reporting-summary-flat.pdf

Life sciences study design

All studies must disclose on these points even when the disclosure is negative.

Sample size	No statistical methods were used to calculate sample size. Sample size were determined based on prior studies and literature in the filed using similar experiments (Vara et al. 2019 Mol Biol Evol; Vara et al. 2019 Cell Reports; Capilla et al. 2014 Proc Biol Sci). A minimum of three mice per diploid number was included per analysis.
Data exclusions	No data was excluded for the analysis.
Replication	Experiments were performed in duplicates, all attempts were successful.
Randomization	No specific randomization method was used, but samples were processed simultaneously to control for potential covariates. Mice were randomly assigned based in their diploid number. Cells for imaging were selected randomly.
Blinding	Investigators were blinded to group allocation during data collection and analysis.

Reporting for specific materials, systems and methods

We require information from authors about some types of materials, experimental systems and methods used in many studies. Here, indicate whether each material, system or method listed is relevant to your study. If you are not sure if a list item applies to your research, read the appropriate section before selecting a response.

Materials & experimental systems

n/a	Involved in the study	
☐	☒	Antibodies
☐	☒	Eukaryotic cell lines
☒	☐	Palaeontology and archaeology
☐	☒	Animals and other organisms
☒	☐	Human research participants
☒	☐	Clinical data
☒	☐	Dual use research of concern

Methods

n/a	Involved in the study	
☒	☐	ChIP-seq
☐	☒	Flow cytometry
☒	☐	MRI-based neuroimaging

Antibodies

Antibodies used	anti-mouse MLH1, BD Pharmingen Cat#551092; anti-rabbit H3K9me3, Abcam Cat#ab8898; anti-rabbit SYCP3, Abcam Cat#ab15093, anti-mouse RAD51, Millipore #PC-130, anti-rabbit Cy3 (Jackson ImmunoResearch Laboratories Cat#111-165-003), anti-mouse FITC

	(Jackson ImmunoResearch Laboratories Cat# 115-095-003), anti-mouse Cy5 (Jackson ImmunoResearch Laboratories Cat# 115-175-008); anti-human CREST serum (non commercial) was kindly provided by M. Fritzler (University of Calgary, Canada)
Validation	anti-mouse MLH1, https://www.bdbiosciences.com/us/applications/research/apoptosis/purified-antibodies/purified-mouse-anti-mlh-1-with-control/p/551092<br><br>anti-rabbit SYCP3, https://www.abcam.com/scp3-antibody-ab15093.html<br><br>anti-rabbit H3K9me3, https://www.abcam.com/histone-h3-tri-methyl-k9-antibody-chip-grade-ab8898.html<br><br>anti-mouse RAD51, https://www.merckmillipore.com/ES/es/product/Anti-Rad51-Ab-1-Rabbit-pAb,EMD_BIO-PC130<br><br>anti-rabbit Cy3, https://www.jacksonimmuno.com/catalog/products/111-165-003<br><br>anti-mouse FITC, https://www.citeab.com/antibodies/2036422-115-095-003-fluorescein-fitc-affinipure-goat-anti<br><br>anti-mouse Cy5, https://www.jacksonimmuno.com/catalog/products/115-175-146

Eukaryotic cell lines

Policy information about cell lines

Cell line source(s)	Primary fibroblast cell line derived from a male mouse from the BRbS system.
Authentication	Authentication by karyotyping.
Mycoplasma contamination	We confirm the cell line tested negative for mycoplasm.
Commonly misidentified lines<br>(See ICLAC register)	No commonly misidentified cell lines were used in the study.

Animals and other organisms

Policy information about studies involving animals; ARRIVE guidelines recommended for reporting animal research

Laboratory animals	House mouse strain (Mus musculus domesticus) used in this study: adult males (from 9 to 18 weeks) C57BL76J mice.Housing conditions: A 14-hour light/10-hour dark cycle or 12 light/12 dark cycle. Temperature of 65-75°F (~18-23°C) with 40-60% humidity.
Wild animals	Mice of Mus musculus domesticus Schwarz and Schwarz, 1943 were live-captured with Sherman traps in the provinces of Barcelona and Tarragona (Catalonia, Spain) in the following locations: Castelldefels, Castellfollit del Boix, Caldes de Montbui, Santa Sandurni d'Anoia and Viladecans. Mice were transferred to Universitat Autonoma de Barcelona and sacrificed through cervical dislocation (CD) euthanasia upon arrival. All mice collected (males and females) were adults (between 9-18 weeks).
Field-collected samples	Field-collected mice correspond to the mice listed in "Wild animals" field above. Capture procedure followed the guidelines and ethical approval of the CEEAH from the Departament d'Agricultura, Ramaderia, Pesca, Alimentació i Medi Natural of the Generalitat de Catalunya (SF/934). All captured animals were immediately transferred to the laboratory and culled following protocols from the CEEAH from UAB (CEEAH 2920). No housing required.
Ethics oversight	Mice were manipulated in compliance with the guidelines of the Ethics Committee on Animal and Human experimentation (CEEAH) from UAB (CEEAH 2920).

Note that full information on the approval of the study protocol must also be provided in the manuscript.

Flow Cytometry

Plots

Confirm that:

☒ The axis labels state the marker and fluorochrome used (e.g. CD4-FITC).

☒ The axis scales are clearly visible. Include numbers along axes only for bottom left plot of group (a 'group' is an analysis of identical markers).

☒ All plots are contour plots with outliers or pseudocolor plots.

☒ A numerical value for number of cells or percentage (with statistics) is provided.

Methodology

Sample preparation	Cell suspension was obtained from mouse testis, in which an immunofluorescence in suspension was performed for specific markers. Cells were dyed vith Hoechst 33342 previous to sorting.

Instrument	BD InfluxTM (BD Biosciences).
Software	BD FACSTM Sortware (version 1.0).
Cell population abundance	Sorted fractions were between 500,000 to 1 M cells. Fraction's purity was evaluated after every FACS experiment by visualizing cells under the microscope, confirming cell type by DAPI morphology using immnufluorescence with specific markers.
Gating strategy	Cells were discriminated from debris by the FSC/SSC profile, then cells were sorted by plotting Hoechst Blue (UV355-460/50) vs. Hoechst red (UV355-670/30) emissions, discriminating them by both their DNA content and their complexity.

☒ Tick this box to confirm that a figure exemplifying the gating strategy is provided in the Supplementary Information.
